# On-Tissue Chemical Derivatization for Comprehensive Mapping of Brain Carboxyl and Aldehyde Metabolites by MALDI–MS Imaging

**DOI:** 10.1021/jasms.2c00336

**Published:** 2023-04-13

**Authors:** Ibrahim Kaya, Luke S. Schembri, Anna Nilsson, Reza Shariatgorji, Sooraj Baijnath, Xiaoqun Zhang, Erwan Bezard, Per Svenningsson, Luke R. Odell, Per E. Andrén

**Affiliations:** # Department of Pharmaceutical Biosciences, Spatial Mass Spectrometry, Science for Life Laboratory, Uppsala University, SE-75124 Uppsala, Sweden; ‡ Department of Medicinal Chemistry, Uppsala University, SE-75123 Uppsala, Sweden; § Section of Neurology, Department of Clinical Neuroscience, Karolinska Institutet, SE-17177 Stockholm, Sweden; ∥ Université de Bordeaux, Institut des Maladies Neurodégénératives, F-33000 Bordeaux, France

## Abstract

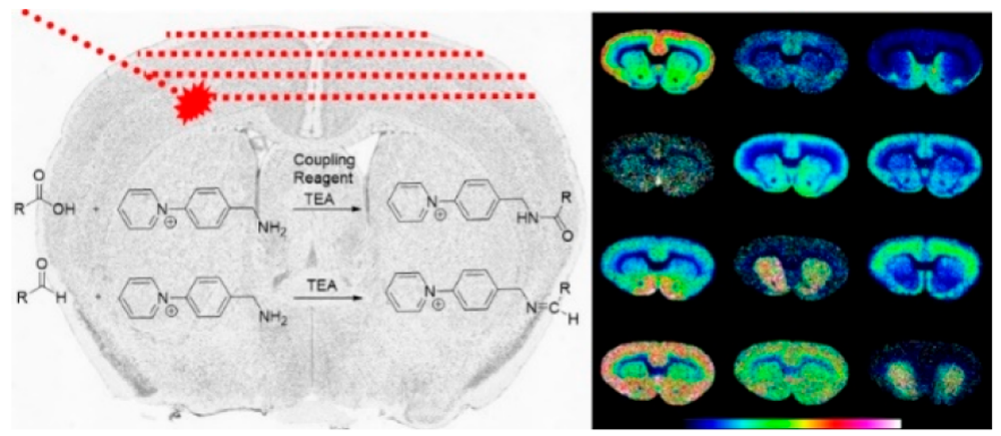

The visualization of small metabolites by MALDI mass spectrometry imaging in brain tissue sections is challenging due to low detection sensitivity and high background interference. We present an on-tissue chemical derivatization MALDI mass spectrometry imaging approach for the comprehensive mapping of carboxyls and aldehydes in brain tissue sections. In this approach, the AMPP (1-(4-(aminomethyl)phenyl)pyridin-1-ium chloride) derivatization reagent is used for the covalent charge-tagging of molecules containing carboxylic acid (in the presence of peptide coupling reagents) and aldehydes. This includes free fatty acids and the associated metabolites, fatty aldehydes, dipeptides, neurotoxic reactive aldehydes, amino acids, neurotransmitters and associated metabolites, as well as tricarboxylic acid cycle metabolites. We performed sensitive ultrahigh mass resolution MALDI-MS detection and imaging of various carboxyl- and aldehyde-containing endogenous metabolites simultaneously in rodent brain tissue sections. We verified the AMPP-derivatized metabolites by tandem MS for structural elucidation. This approach allowed us to image numerous aldehydes and carboxyls, including certain metabolites which had been undetectable in brain tissue sections. We also demonstrated the application of on-tissue derivatization to carboxyls and aldehydes in coronal brain tissue sections of a nonhuman primate Parkinson’s disease model. Our methodology provides a powerful tool for the sensitive, simultaneous spatial molecular imaging of numerous aldehydes and carboxylic acids during pathological states, including neurodegeneration, in brain tissue.

## Introduction

Carboxyl and aldehyde functional groups are a common feature of various biomolecules, including free fatty acids, eicosanoids, amino acids and associated metabolites, keto acids, peptides, bile acids, neurotoxic aldehydes, neurotransmitters, fatty aldehydes, and polycarboxylic acids. They are also prevalent in numerous key metabolic pathways in the brain, such as the tricarboxylic acid cycle (TCA), kynurenine pathway, serotonin and dopamine neurotransmitter metabolism, glycolysis, lipolysis and lipid peroxidation, along with the metabolism and regulation of both amino acids and short-chain fatty acids.^1−3^ Moreover, molecules containing carboxyls and aldehydes play critical roles in biological systems.^2,4^ Brain region-specific disruptions associated with carboxyls and aldehydes are involved in a wide range of pathological processes, namely, essential fatty acid disorders,^5^ peroxisomal disorders,^6^ cancer,^7^ and neurodegenerative disorders^8^ such as Alzheimer’s disease,^4,9^ and Parkinson’s disease (PD).^10^

Matrix-assisted laser desorption/ionization mass spectrometry imaging (MALDI-MSI) offers high-throughput spatial determination of the relative abundances of endogenous biomolecules, including metabolites,^11^ lipids,^12^ peptides,^13^ and proteins,^14^ along with exogenous pharmaceutical compounds.^15^ However, the detection of many aldehydes is challenging due to the low ionization efficiency of these compounds, while the detection of certain carboxyls within tissue sections is challenging due to low overall abundances. Therefore, advanced imaging approaches for carboxyls and aldehydes in the brain are needed to better understand the metabolic processes and disorders relevant to neuroscience.

On-tissue chemical derivatization is a well-established strategy for increasing the selectivity and sensitivity of mass spectrometry imaging toward low abundance, poor-ionizing nonpolar and low polarity compounds.^16−22^ The (1-(4-(aminomethyl)phenyl)pyridin-1-ium chloride (AMPP) derivatization reagent has shown promise for efficient derivatization via amide linkage and sensitive detection of carboxyl-containing biomolecules using LC–MS^23^ and MALDI-MS^24^ in positive ionization mode. Given the presence of a nucleophilic primary amine, we reasoned that AMPP should undergo Schiff base reactions with aldehyde-containing biomolecules under these conditions. The developed method could provide an unprecedented approach for the simultaneous detection of carboxyl and aldehyde metabolites. Herein, we present the development of AMPP as a dual-purpose reagent for the on-tissue derivatization of numerous carboxyl- and aldehyde-containing endogenous metabolites, including previously undetectable analytes, in brain tissue sections from rat and primate models of PD.

We demonstrated that our methodology can successfully delineate distinct alterations in carboxyl- and aldehyde-containing biomolecules within key metabolic pathways in brain tissue sections from rat and primate models of PD.

## Methods

### Chemicals and Reagents

All of the chemicals used in matrix and solvent preparation were of pro-analysis grade and obtained from Sigma-Aldrich (St. Louis, MO) unless otherwise specified. AMPP (1-(4-(aminomethyl)phenyl)pyridine-1-ium chloride (97+%) was purchased from Active Scientific (Prien am Chiemsee, Germany).

### Animal Experiments

Untreated (*n* = 2) or unilaterally 6-hydroxydopamine (6-OHDA)-lesioned (*n* = 2) male Sprague–Dawley rats (150–200 g) were used for brain tissue sampling. Experiments were performed in agreement with the European Communities Council Directive of November 24, 1986 (86/609/EEC) on the ethical use of animals and were approved by the local ethical committee at the Karolinska Institute (N350/08 and N105/16). The unilaterally lesioned rats were injected with 6-OHDA (2.5 μL of a 5 mg/mL solution) into the median forebrain bundle of the right hemisphere (anterior-posterior, 2.8 mm; medial-lateral, 2.0 mm; and ventral, 9.0 mm). Two weeks after the unilateral 6-OHDA lesioning, the rats were injected with apomorphine (1 mg/kg, i.p.) to check for rotational behavior. The rotation tests were measured by Rotorat software (Med Associates, Inc., Fairfax, VT) for 30 min. Rats were sacrificed 8 days after the apomorphine administration. The brains of animals were extracted, snap-frozen with dry ice-cooled isopentane, and kept at −80 °C until sectioning. Coronal brain sections (12 μm) were sectioned using a cryostat (Leica CM 1900; Leica Microsystems, Wetzlar, Germany) at −20 °C and thaw-mounted onto indium tin oxide-coated glass slides (Bruker Daltonics, Bremen, Germany) and stored at −80 °C before MALDI-MSI analysis.

Primate experiments were performed using tissue originating from a previously published brain bank.^25−27^ Following the acceptance of the study design by the Institute of Lab Animal Science (Chinese Academy of Science, Beijing, China), experiments were carried out in an AAALAC-accredited facility in accordance with the European Communities Council Directive of November 24, 1986 (86/609/EEC) for the care of laboratory animals. Briefly, tissue from two female rhesus monkeys (Macaca mulatta, Xierxin; mean age = 5 ± 1 years; mean weight = 5.3 ± 0.8 kg) was used in the present study. One of the animals served as a control; therefore, it did not receive any treatment. The other animal was administered 1-methyl-4-phenyl-1,2,3,6-tetrahydropyridine (MPTP) (0.2 mg/kg, administered intravenously) on a daily basis, in line with a previously published protocol.^28,29^ Following stabilization of the MPTP-induced syndrome, animal behavior was assessed as suggested in previous research.^25,30,31^ Animals were killed by sodium pentobarbital overdose (150 mg/kg, administered intravenously), after which the brains were quickly removed, immediately frozen by immersion in −45 °C isopentane, and stored at −80 °C. Coronal brain tissue sections (−7.5 mm from anterior commissure)^32^ from the control and MPTP-lesioned animals were cut at 12 μm thickness using a cryostat (CM3050S; Leica Microsystems) at −20 °C, thaw-mounted onto indium tin oxide–coated glass slides (Bruker Daltonics) and stored at −80 °C before MALDI-MSI analysis.

### Sample Preparation and Application of Derivatization Reagents

In order to compare coupling reagents for active ester formation, equimolar (2 mM) solutions of EDCl (3-(dimethylamino)propyl)ethyl carbodiimide hydrochloride), HATU (*O*-(7-azabenzotriazol-1-yl)-1,1,3,3-tetramethyluronium hexafluorophosphate), HBTU (*O*-(benzotriazol-1-yl)-1,1,3,3-tetramethyluronium hexafluorophosphate), TOTU (*O*-[cyano(ethoxycarbonyl)methyleneamino]-*N*,*N,N′,N’*-tetramethyluronium tetrafluoroborate), COMU (1-[(1-(cyano-2-ethoxy-2-oxoethylideneaminooxy)dimethylaminomorpholinomethylene)]methanaminium hexafluorophosphate), PyAOP ([(7-azabenzotriazol-1-yl)oxy]tris(pyrrolidino)phosphonium hexafluorophosphate), PyOxim (*O*-[(cyano(ethoxycarbonyl)methyliden)amino]yloxytri(pyrrolidino)phosphonium hexafluorophosphate), PyBOP (benzotriazol-1-yloxytri(pyrrolidino)phosphonium hexafluorophosphate) were prepared in glass vials in 70% acetonitrile solution. Each solution contained 2 mM of triethylamine (TEA). Standard solutions (1 mg/mL) of 4-carboxybutyltriphenylphosphonium bromide and *N*-acetylaspartic acid were prepared in 50% EtOH and diluted by a factor of 4. For each coupling reagent, the diluted 4-carboxybutyltriphenylphosphonium bromide solution was spotted (9 × 0.5 μL) on a MTP 384 ground steel target plate (Bruker Daltonics) and left to dry at room temperature. Next, heated solutions of coupling reagents were sprayed over the spotted standards using an automated pneumatic sprayer (HTX Technologies LLC, Chapel Hill, NC) combined with an HPLC pump (Dionex, Sunnyvale, CA). The pump was kept running at 80 μL/min using a 50% acetonitrile (ACN) pushing solvent with isocratic pressure before the experiments. The solutions were sprayed using the following instrumental parameters: solvent flow rate of 80 μL/min at isocratic pressure; nitrogen pressure of 6 psi; spray temperature of 80 °C; 10 passes (all horizontal); nozzle spray velocity of 1100 mm/min; and track spacing of 2.0 mm. In the following step, 5 mg/mL norharmane (dissolved in 80% MeOH solution) was sprayed over the MTP target plate using a HTX automated pneumatic sprayer, which was combined with a pump (AKTA FPLC P-905 pump, Amersham Pharmacia Biotech, Amersham, UK) to spray the heated matrix solution over the spotted standards. Prior to the experiments, the pump was kept running at 70 μL/min using a 50% ACN pushing solvent with isocratic pressure. The matrix solution was sprayed using the following instrumental parameters: solvent flow rate of 70 μL/min at isocratic pressure; nitrogen flow of 6 psi; spray temperature of 65 °C; 10 passes (all horizontal); nozzle spray velocity of 1200 mm/min; and track spacing of 2.0 mm. In order to compare coupling reagents for charge-tagged amide formation, the same experiments were performed except for the difference that each coupling reagent solution contained 2.5 mM AMPP derivatization reagent.

Glass slides with consecutive brain tissue sections were desiccated at room temperature for 20 min before the application of the derivatization and matrix solutions. Solutions of four coupling reagents (HATU (2 mM), HBTU (2 mM), EDCl (2 mM), and TOTU (2 mM), each of which contained the AMPP (2.5 mM) derivatization reagent) and TEA (1.5 mM), were dissolved in 70% ACN in a glass vial and briefly sonicated. The solutions were sprayed using the previously described instrumental parameters, with the exception that 6, 8, 10, or 12 passes (all horizontal) were conducted on four direct consecutive coronal rat brain tissue sections. This was followed by spraying 5 mg/mL norharmane (dissolved in 80% MeOH solution) over the tissue sections using the same instrumental parameters that were applied to the MTP target plate (see above).

For AMPP derivatization of primate brain samples, the control and MPTP primate coronal brain tissue sections were prepared using the same sample preparation protocol (with 6 passes) as described above.

To compare how the AMPP derivatization improved the detection of target compounds, consecutive brain tissue sections were coated with 9-aminoacridine (9-AA), 2,5-dihydroxybenzoic acid (DHB), and AMPP (coupled with HATU). 9-AA (5 mg/mL) was dissolved in 80% MeOH and briefly sonicated. The heated solution was sprayed over the tissue sections using the following instrumental parameters: solvent flow rate of 70 μL/min at isocratic pressure; nitrogen flow of 6 psi; spray temperature of 75 °C; 6 passes; nozzle head velocity of 1200 mm/min; and track spacing of 2.0 mm. DHB (35 mg/mL) was dissolved in 50% ACN (with 0.2% TFA) and briefly sonicated. The heated solution was sprayed over the tissue sections using the following instrumental parameters: solvent flow rate of 70 μL/min at isocratic pressure; nitrogen flow of 6 psi; spray temperature of 95 °C; 6 passes; nozzle head velocity of 1200 mm/min; and track spacing of 2.0 mm.

### MALDI–MS and MALDI–MS/MS Profiling and Imaging Analysis

All of the MALDI-MSI experiments were performed in positive or negative ionization mode using a MALDI Fourier-transform ion cyclotron resonance (FTICR) (Solarix XR 7T-2ω, Bruker Daltonics) mass spectrometer equipped with a Smart-beam II 2 kHz laser. Mass resolution was calculated as ∼260,000 at *m*/*z* 350 during the MSI analysis of the derivatized molecules on brain tissue sections. Laser power was optimized at the start of each run and then held constant during the MALDI–MSI experiment. Laser size was chosen to give a lateral resolution of 100 or 20 μm for high-lateral resolution analysis. For on-tissue derivatization and MALDI-MSI analysis, the instrument was tuned for the optimal detection of derivatized metabolites (*m*/*z* 200–1000) in positive-ion mode using the quadrature phase detection (QPD) (2ω) mode. The time-of-flight (TOF) values were set at 0.5 ms, and the transfer optics frequency was kept at 4 MHz for both polarity analyses. The quadrupole isolation *m*/*z* value (Q1 mass) was set at *m*/*z* 250 for both polarity analyses. Spectra were collected by summing 100 laser shots per pixel in both polarities. All of the methods were calibrated externally with red phosphorus over an appropriate mass range. The ion signal at a *m*/*z* value of 368.199548 (AMPP cluster ion) was used for the internal calibration of derivatization experiments.

We performed dual polarity (i.e., both positive and negative ionization modes) MALDI-MSI on the same spots of the MALDI steel MTPs at 250 μm resolution to evaluate active ester and amide formation from carboxylic acids. First, we performed a positive polarity analysis to detect charge-tagged amide molecules and carboxybutyltriphenylphosphonium, after which a negative polarity analysis of carboxylic acids was performed with an offset value of 125 μm in the spots. The ion signal at a *m*/*z* value of 167.061472 (monoisotopic peak of norharmane matrix [M – H]^−^) was used for the internal calibration of the negative polarity analysis, while a *m*/*z* value of 362.143018 (monoisotopic peak of 4-carboxybutyltriphenylphosphonium [M]^+^) was used for the internal calibration of the positive polarity analysis. For the negative-ion mode analysis using the 9-AA matrix, the instrument was tuned for the optimal detection of metabolites (*m*/*z* 107–1000 Da or *m*/*z* 43–500) using the QPD (2ω) mode. The TOF values were set at 0.5 or 0.4 ms (for *m*/*z* ≤ 100), and the transfer optics frequency was kept at 4 MHz. The quadrupole isolation *m*/*z* value (Q1 mass) was set as *m*/*z* 107 or *m*/*z* 43 (for *m*/*z* ≤ 100). The ion signal at a *m*/*z* value of 193.077122 (monoisotopic peak of 9-AA matrix [M – H]^−^) was used for the internal calibration of the negative polarity analysis. For the positive-ion mode analysis using the DHB matrix, the instrument was tuned for the optimal detection of metabolites (*m*/*z* 107–1000) using the QPD (2ω) mode. The TOF values were set at 0.5 ms, and the transfer optics frequency was kept at 4 MHz. The quadrupole isolation *m*/*z* value (Q1 mass) was set as *m*/*z* 107. The ion signal at a *m*/*z* value of 273.039364 (monoisotopic peak of DHB matrix cluster [2M – 2H_2_O + H]^+^) was used for the internal calibration of the positive polarity analysis. Laser size was chosen to give a lateral resolution of 100 μm, and spectra were collected by summing 100 laser shots per pixel in both polarities for 9-AA and DHB MALDI-MSI analysis.

### Identification of Metabolites

The ions that displayed distributions within brain tissue sections were primarily identified by database searches for previously detected metabolites, e.g., www.hmdb.ca(33) and www.lipidmaps.org,^34^ and previously published studies based on the high mass accuracy (1–2 ppm) and fine isotopic structure provided by MALDI-FTICR MS analysis. Predicted molecular formula calculations were performed using ChemCalc software (www.chemcalc.org^35^). MALDI-MS/MS was performed on tissue sections by collecting several spectra with the smallest possible precursor mass window width from brain regions where the target ion is abundant after MALDI-MSI experiments using MALDI-collision-induced dissociation (CID)-FTICR; the identified product ions were then compared to the product ion spectra of available standards, previously published studies and/or interpreted fragmentation pathways of derivatized molecules (SI Figures S1–3). In the case of MS/MS imaging, the brain tissue distributions of product ions were compared to the distribution of the corresponding precursor ion (see examples in SI Figures S4 and S5). The distributions of validated metabolites from previously published studies, particularly those of neurotransmitters and associated metabolites containing phenolic hydroxyls and primary amine groups, were also considered when identifying metabolites; this included distributions and unilateral changes of neurotransmitters and the associated metabolites within 6-OHDA-lesioned rat and MPTP macaque brain tissue sections^18^ (SI Figure S6). In the case that ions were present at low abundances in the brain tissue section, identification was based on mass accuracy and the annotations of the metabolites were made based on the possible abundance within brain tissue sections or what had been reported in previously published studies (SI Table S1). The selectivity of the AMPP derivatization reagents towards aldehydes and/or carboxyls was used to identify species bearing such functional groups. Since AMPP derivatization adds nitrogen atoms to the derivatized aldehyde- and/or carboxyl-containing molecule, the nitrogen rule was also considered for the identification of derivatized metabolite species. In order to evaluate whether derivatization with AMPP/HATU significantly impacts the distribution of metabolites, we compared [M – H]^−^ ion images of abundant carboxylic acids, e.g., aspartic acid and *N*-acetyl aspartic acid, with the corresponding AMPP/HATU-derivatized ion images (SI Figure S7).

### Data Processing

MSI data were visualized in FlexImaging (v. 5.0, Bruker Daltonics). For further analysis, data were imported to SCiLS Lab (v. 2019a Pro, Bruker Daltonics). In the rat brain tissue images, brain regions were annotated according to a stereotaxic atlas.^36^ To evaluate the formation of active esters, the average peak areas of the monoisotopic peaks ([M]^+^) of charge-tagged active ester ions at *m*/*z* 480.183 (from OBt-based PyBOP and HBTU coupling reagents), *m*/*z* 481.515 (from OAt-based PyAOP and HATU coupling reagents), *m*/*z* 487.178 (from Oxyma-based TOTU, PyOxim and COMU coupling reagents), and *m*/*z* 518.293 (from the carbodiimide-based EDCl coupling reagent) were calculated, after which their ratio to the average peak area of the precursor charge-tagged carboxylic acid signal at *m*/*z* 362.143 (monoisotopic peak of 4-carboxybutyltriphenyl-phosphonium [M]^+^) was determined to reflect the conversion of carboxylic acid to active ester. To evaluate charge-tagged amide formation, the average peak areas of the monoisotopic peaks of singly charged amide molecule at *m*/*z* 530.248 (from carboxybutyltriphenylphosphonium) and *m*/*z* 342.144 (from *N*-acetylaspartic acid) were calculated, after which their ratios to the average peak areas of the precursor charge-tagged carboxylic acid signal at *m*/*z* 362.143 (monoisotopic peak of 4-carboxybutyltriphenylphosphonium [M]^+^) and *N*-acetylaspartic acid at *m*/*z* 174.040 (monoisotopic peak of *N*-acetylaspartic acid [M – H]^−^), respectively, were evaluated to determine the conversion of carboxylic acid to a charge-tagged amide.

## Results and Discussion

### Improved Detection of Carboxyl and Aldehyde Metabolites by On-Tissue Chemical Derivatization Using AMPP

**Figure 1:**
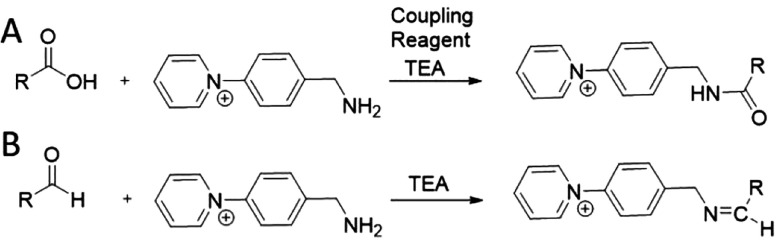
Schematic of the reaction between the AMPP derivatization reagent and carboxylic acids or aldehydes. (A) Reactions with carboxylic acids, in the presence of peptide coupling reagents, and (B) aldehydes yield covalently bound charged-tagged amides and Schiff bases, respectively. TEA: triethylamine.

The reaction of AMPP with carboxyls requires coupling reagents to mediate the formation of an active ester, which can react with the amine group in AMPP to form a charge-tagged amide molecule (Figure 1A). In contrast, AMPP is expected to react directly with aldehydes through a Schiff base reaction (Figure 1B). Thus, we first sought to identify suitable reaction conditions for the simultaneous derivatization of carboxyls and aldehydes using AMPP.

**Figure 2:**
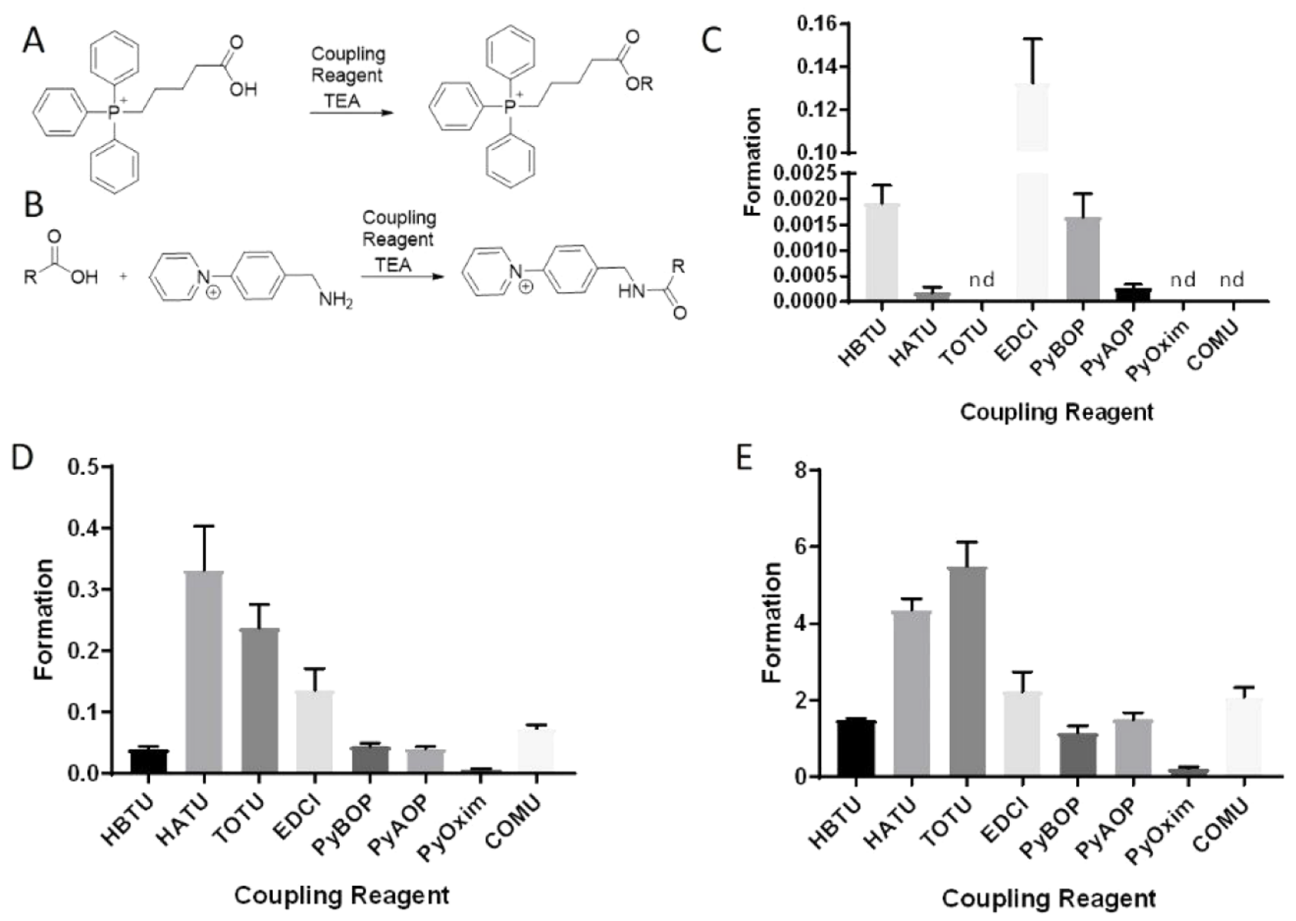
Evaluation of eight different coupling reagents for active ester and amide formation from carboxylic acids using AMPP derivatization, analyzed by MALDI-MSI. (A) Schematic of the reaction between 4-carboxybutyltriphenylphosphonium and peptide coupling reagents (PyAOP, PyBOP, HATU, HBTU, PyOxim, TOTU, COMU, EDCl), which yields OBt- (from PyBOP and HBTU), OAt- (from PyAOP and HATU), ethyl 2-cyano-2-(hydroxyimino)acetate (Oxyma)- (from TOTU, PyOxim and COMU), or carbodiimide-based (from EDCl) active ester compounds with a permanent positive charge. (B) Schematic of the reaction between carboxylic acids (e.g., 4-carboxybutyltriphenylphosphonium bromide and *N*-acetylaspartic acid) and AMPP in the presence of peptide coupling reagents, which yields charge-tagged amide compounds with a permanent positive charge. (C) The bar graph (*n* = 9) indicates active ester formation from 4-carboxybutyltriphenylphosphonium as the ratio of the signal of formed active ester to the signal of 4-carboxybutyltriphenylphosphonium in the presence of eight different peptide coupling reagents. (D) The bar graph (*n* = 9) indicates amide formation from carboxylic acid as the ratio of the signal of formed charged-tagged amides to the signal of 4-carboxybutyltriphenylphosphonium formed via the reaction between 4-carboxybutyltriphenylphosphonium and AMPP in the presence of eight different peptide coupling reagents. (E) The bar graph (*n* = 9) indicates amide formation from carboxylic acid as the ratio of the signal of formed charge-tagged amides to the signal of *N*-acetyl aspartic acid formed via the reaction between *N*-acetyl aspartic acid and AMPP in the presence of eight different peptide coupling reagents. TEA, triethylamine; nd, not detected.

A number of peptide coupling reagents exist for fast and efficient coupling reactions which produce amides from carboxyls.^37^ To investigate the peptide coupling reactions with AMPP under MALDI-MSI conditions, we selected eight such reagents (PyBOP, HBTU, PyAOP, HATU, TOTU, PyOxim, COMU, and EDCI) to evaluate both the formation of active esters and charge-tagged amides under MALDI-MSI conditions (Figure 2A,B). This is because the final charge-tagged amide product depends upon both the formation and reaction of the active ester during peptide coupling reactions.

A series of screening reactions were initially conducted to evaluate the carboxylic acid conversion of a corresponding active ester under standard MALDI-MSI conditions. To enable detection of the carboxylic acid, permanently charged 4-carboxylbutyltriphenylphosphonium was used as a model substrate (Figure 2A). This compound was spotted on a MALDI multitarget plate and equimolar solutions of the eight different coupling reagents were sprayed onto the plate, after which a norharmane matrix solution was sprayed with a temperature-controlled pneumatic sprayer. MALDI-MSI analysis of the standard solution spots was performed, and the ratio of active ester to 4-carboxybutyltriphenylphosphonium was used to estimate conversion (Figure 2C). The carbodiimide-based (from EDCl) coupling reagent produced the highest active ester formation, and the OBt-based (from PyBOP and HBTU) coupling reagents were more effective than OAt-based (from PyAOP and HATU) coupling reagents (Figure 2C).

Interestingly, the active esters were not detected when Oxyma-based (from TOTU, PyOxim and COMU) coupling reagents were used (Figure 2C). To evaluate charge-tagged amide formation, a standard solution of 4-carboxylbutyltriphenylphosphonium and AMPP was spotted on a MALDI multitarget plate together with the aforementioned coupling reagents. In this case, the ratio of charged-tagged amide to 4-carboxybutyltriphenylphosphonium (Figure 2D) was used to compare the different reagents. The highest product signal ratios were achieved when HATU and TOTU were used as peptide coupling reagents (Figure 2D), with *N*-acetylaspartic acid yielding similar results (Figure 2E). This suggests that although there is lower formation of active ester, the more efficient reaction with AMPP produces larger amounts of charge-tagged amide. While the active ester from oxyma-based coupling reagents (TOTU, PyOxim and COMU) was not detected with MALDI-MSI, TOTU offered a high ratio of the charge-tagged amide. Interestingly, EDCl demonstrated the highest active ester formation; however, the charge-tagged amide signal was relatively low, which suggests that the active ester formed using EDCl has low reactivity.

Having identified the four most promising coupling reagents (HATU, TOTU, HBTU, and EDCl), the next step was to test their efficiency in on-tissue chemical derivatization experiments. The solutions of TOTU, EDCl, HATU and HBTU, which contained equimolar AMPP, were sprayed using 6, 8, 10, or 12 passes over consecutive rat brain tissue sections, after which norharmane matrix solution was sprayed over the tissue sections. Norharmane was chosen as the matrix solution for MALDI-MSI analysis of derivatized carboxyls and aldehydes for several reasons. First, most commercial MALDI matrices, such as 1,5-diaminonapthelene (1,5-DAN), 9-AA, α-cyano-4-hydroxycinnamic acid, and DHB, contain either carboxylic acid or primary amine groups in the molecular structure. This may cause side reactions with either the coupling reagents, the resulting active esters, or the endogenous aldehydes within peptide coupling reactions, which will decrease the MALDI-MSI detection of derivatized aldehyde and carboxylic acid molecules as well as complicate the spectra. The molecular structure of norharmane, on the other hand, does not contain carboxylic acid or primary amine groups and is an efficient MALDI matrix for both negative- and positive-ion MALDI-MSI analysis.^38^ Furthermore, norharmane is a basic molecule which can stimulate the formation of active ester, along with the formation of subsequent charge-tagged amides of carboxylic acids and charge-tagged Schiff base molecules of aldehydes under the MALDI-MSI sample preparation conditions. Therefore, norharmane is an ideal matrix for the MALDI-MSI analysis of AMPP-derivatized carboxyls and aldehydes in tissue sections.

The identification of metabolites was performed directly in the brain tissue section with on-tissue MALDI-CID-FTICR MS/MS (SI Figures S1–3) and/or MS/MS imaging (SI Figures S4 and S5) with high mass accuracy (SI Table S1).

In a previous report regarding the on-tissue chemical derivatization of carboxylic acids, Sun et al. used 1,5-DAN as a matrix to desorb *N*,*N*,*N*-trimethyl-2-(piperazin-1-yl)ethan-1-aminium iodide (TMPA) derivatized carboxylic acids.^39^ However, primary amines in the molecular structure of 1,5-DAN compete with the TMPA derivatization of active esters to prevent the detection of low-abundance carboxylic acids. Indeed, the authors only report the detection of abundant carboxylic acids in the tissue sections.^39^

**Figure 3:**
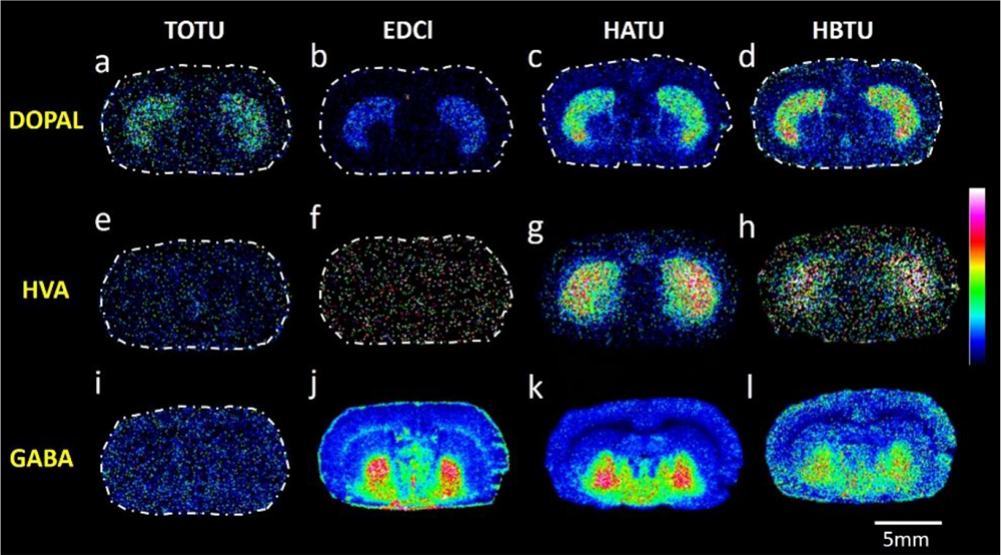
MALDI-MS images of carboxyl- and aldehyde-containing metabolites acquired from a rat brain tissue section. Images A–D illustrate the distribution of DOPAL, images E–H illustrate the distribution of HVA, and images I–L demonstrate the distribution of GABA; in all cases, AMPP is employed as the derivatization agent in the presence of four different peptide coupling reagents (TOTU, EDCl, HATU, and HBTU). MALDI–MSI experiments were performed on direct consecutive sections for each coupling reagent. Data are shown using a rainbow scale (ion intensity scale) for optimal visualization. Lateral resolution, 100 μm.

To evaluate the on-tissue chemical derivatization performance of the coupling reagents, we compared the MALDI ion images of 4-dihydroxyphenylacetaldehyde (DOPAL), homovanillic acid (HVA) and γ-aminobutyric acid (GABA) (Figure 3), which are endogenous low ionizing and/or low-abundant aldehyde or carboxylic acid molecules in rodent brain tissue sections. HATU and HBTU were useful for the simultaneous derivatization and MALDI-MSI of DOPAL, HVA, and GABA, while EDCl was effective for GABA and DOPAL. TOTU was not useful as a coupling reagent for carboxylic acids under on-tissue chemical derivatization and MALDI-MSI conditions but was adequate for the derivatization and detection of DOPAL. This proves that AMPP coupled with HATU or HBTU can be used to derivatize aldehydes and carboxyls within tissue sections for subsequent MALDI-MSI. The comparison of MALDI-MSI results for consecutive tissue sections that had undergone 9-AA and DHB analysis, and AMPP/HATU derivatization of carboxyls and aldehydes revealed that AMPP/HATU derivatization improved sensitivity toward carboxyls; this significantly improved the MALDI-MSI image quality of certain metabolites when compared to the images obtained using 9-AA (SI Figure S6). We did not observe aldehydes and carboxylic acids as [M + H]^+^ ions when using DHB; therefore, these data are not shown.

Furthermore, AMPP/HATU derivatization allowed the detection of carboxyls which are not detectable as [M – H]^−^ or [M + H]^+^ ions when using 9-AA and DHB in negative- and positive-ion mode, respectively. DOPAC, HVA, 5-hydroxyindoleacetic acid (5-HIAA), γ-hydroxybutyric acid (GHB), pantothenic acid, acetic acid, α-ketoglutaric acid, and oxaloacetic acid are examples of low-abundance carboxyls that were detected using the AMPP/HATU derivatization strategy. The improved detection was more pronounced for aldehyde derivatization with AMPP, as we did not observe any aldehyde molecules such as [M – H]^−^ or [M + H]^+^ ions with either 9-AA in negative ion mode or DHB in positive-ion mode. In contrast, the AMPP/HATU derivatization strategy enabled the detection of aldehyde molecules, e.g., DOPAL, 5-hydroxyindoleacetaldehyde (5-HIAL), acrolein, glucose/galactose, and fatty aldehydes (e.g., pentadecanal and heptadecanal) within brain tissue sections (SI Figure S6, SI Table S1).

We also evaluated whether the AMPP/HATU derivatization method is relevant for the quantification of metabolites. We demonstrated that our method succeeded in quantifying HVA within the striatum of coronal rat brain tissue sections (see SI Figure 8 for a detailed explanation of the quantification experiment).

### Identification of Carboxyl and Aldehyde Metabolites in Different Metabolic Pathways

**Figure 4:**
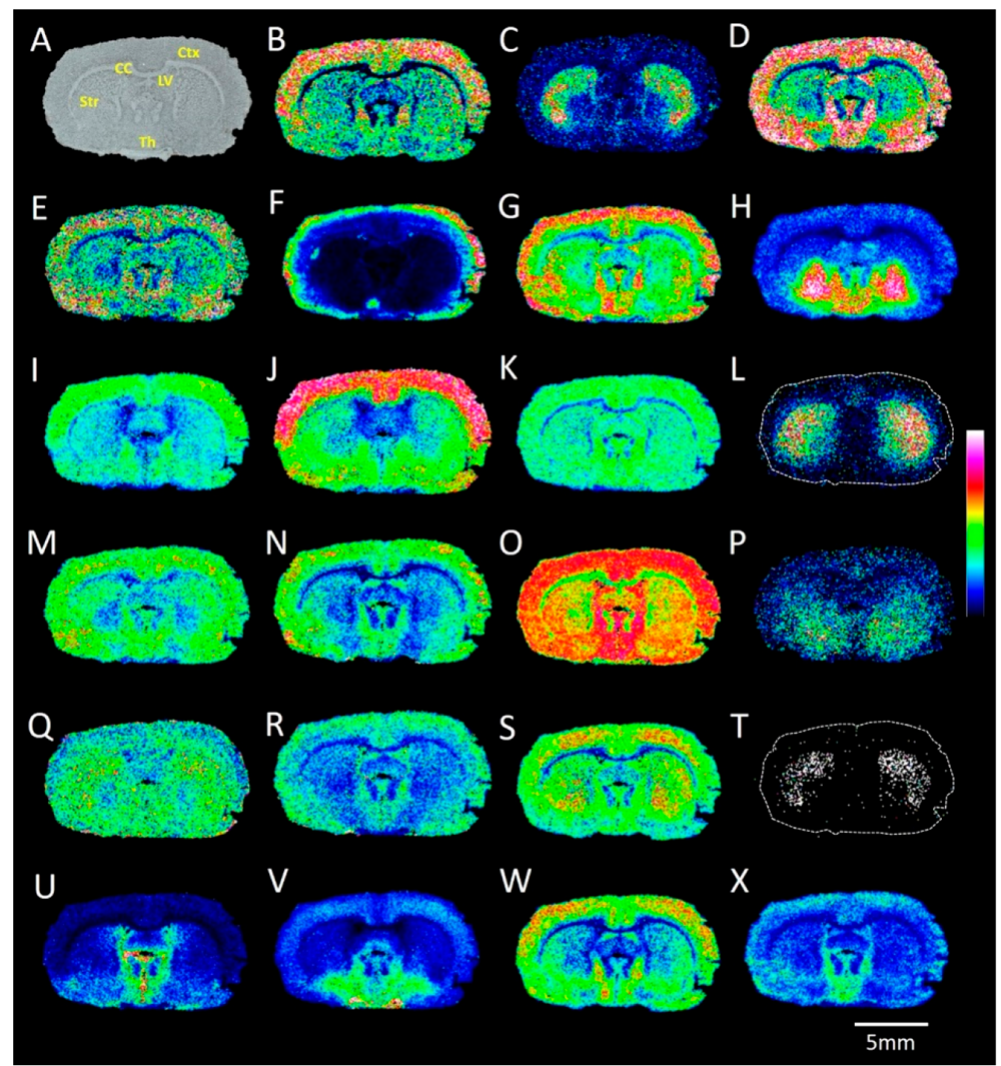
MALDI-MS images of carboxyl- and aldehyde-containing metabolites acquired from a rat brain tissue section. (A) Optical image of a coronal rat brain tissue section. The ion images reveal the distributions of AMPP-derivatized (with HATU as the coupling reagent) endogenous metabolites: (B) fumaric acid; (C) DOPAL; (D) oxaloacetic acid; (E) α-ketoglutaric acid; (F) succinic acid; (G) acetic acid; (H) GABA; (I) *N*-acetyl aspartic acid; (J) aspartic acid; (K) pyruvic acid; (L) HVA; (M) glutamate; (N) citric acid/isocitric acid; (O) lactic acid; (P) 5-HIAA; (Q) glutamine; (R) aconitic acid; (S) malic acid; (T) DOPAC; (U) pantothenic acid; (V) NAAG; (W) glutamate semialdehyde; (X) succinic semialdehyde. The MALDI–MSI experiments were performed independently using different rat brain samples (*n* = 4), all of which produced similar results. Ctx, cortex; CC, corpus callosum; Str, striatum; Th, thalamus; LV, lateral ventricle. Data are shown using a rainbow scale (ion intensity scale) for optimal visualization. Lateral resolution, 100 μm.

The simultaneous detection of carboxyls and aldehydes is advantageous for probing a number of metabolic pathways within brain tissue sections. The TCA cycle, glycolysis, amino acid, and neurotransmitter metabolism, along with lipid synthesis and lipid peroxidation, all represent metabolic pathways that contain a number of biomolecules with carboxyl and/or aldehyde functional groups.^2,5,40^ We have previously presented derivatization techniques which allow the on-tissue derivatization and highly sensitive detection via MALDI-MSI of molecules containing phenolic hydroxyls and primary amines, i.e., various neurotransmitters and the associated metabolites.^18,19,41^ The presented method can detect neurotransmitters and the associated metabolites which contain aldehyde and/or carboxyl groups in the molecular structure in a way that complements our previous methods and increases the number of metabolites that can be detected within brain tissue sections. For instance, the present method enables the detection and imaging of GABA, DOPAC, 5-HIAA and DOPAL; this is valuable because one experiment can be conducted to detect aldehydes and carboxyls with low abundance and/or low ionization efficiency (Figure 4). As such, we could detect various carboxyls and aldehydes, along with some NTs and the associated metabolites involved in serotonergic, GABAergic, and dopaminergic metabolism, in the same experiment. For example, GHB, which is a neurotransmitter and precursor to GABA, glutamate, glycine, and succinic semialdehyde, can be detected together with GABA in the same experiment. Furthermore, pantothenic acid (vitamin B_5_), which is involved in the synthesis of multiple neurotransmitters,^42^ and the dipeptide neurotransmitter *N*-acetylaspartylglutamate (NAAG),^43^ can be detected along with other NTs in the same experiment (Figure 4). Therefore, AMPP/HATU derivatization complements our previous techniques^18,41,44^ through the ability to derivatize carboxyl and/or aldehyde metabolites which do not have phenolic hydroxyls or primary amines in the molecular structure. This is relevant because the distributions of several metabolites, including pantothenic acid, aconitic acid, NAAG, GHB, succinic semialdehyde, acrolein, acetic acid, oxaloacetic acid, α-ketoglutaric acid, and glutamate semialdehyde, have not previously been reported in brain tissue sections (Figure 4, SI Figure S6).

Furthermore, the present approach enables the detection of the distributions of several carboxyl-containing molecules within the TCA cycle, including citric acid/isocitric acid, oxaloacetic acid, malic acid, fumaric acid, α-ketoglutaric acid, aconitic acid, and succinic acid (SI Figure S9). Previous methods using a commercial matrix have only been able to detect a few of the metabolites of the TCA cycle on brain tissue sections.^45,46^ Additionally, the presented approach can detect amino acids, including glutamate, glutamine, aspartic acid, and *N*-acetylaspartic acid, and glutamate semialdehyde, along with pyruvic acid and lactic acid, which are the end products of aerobic and anaerobic glycolysis, respectively, and acetate, which is involved in the TCA cycle.^40^

**Figure 5:**
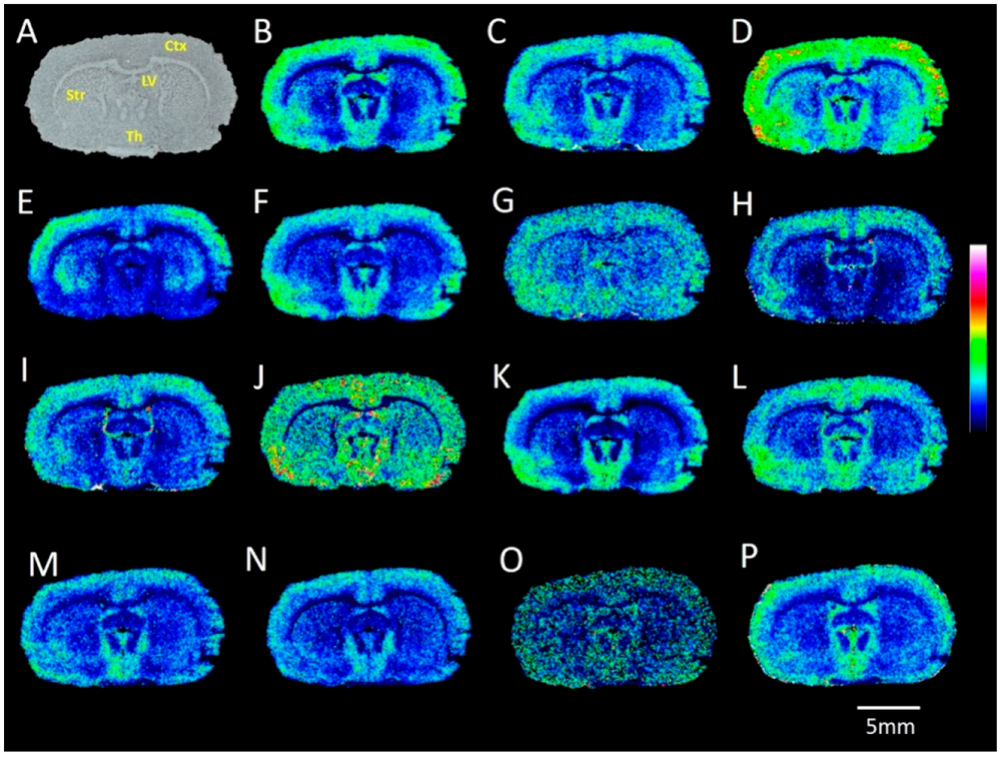
MALDI-MS images of carboxyl and aldehyde metabolites acquired from a rat brain tissue section. (A) Optical image of coronal rat brain tissue section. Ion images reveal the distributions of AMPP-derivatized (with HATU as the coupling reagent) endogenous metabolites: (B) FA (16:0); (C) FA (16:1); (D) FA (18:1); (E) docosahexaenoic acid (DHA); (F) arachidonic acid (AA); (G) FA (20:1); (H) FA (22:5); (I) FA (18:2); (J) FA (20:5); (K) FA (18:0); (L) FA (22:4); (M) acrolein; (N) malondialdehyde/pyruvaldehyde; (O) HNE; (P) pentadecanal. Ctx, cortex; Str, striatum; Th, thalamus; LV, lateral ventricle. The MALDI–MSI experiments were performed independently using different rat brain sections (*n* = 4), all of which produced similar results. Data are shown using a rainbow scale (ion intensity scale) for optimal visualization. Lateral resolution, 100 μm.

Another advantage of AMPP derivatization is specificity toward free fatty acids (FFAs), which was recently demonstrated in an LC–MS study.^24^ FFAs within the brain are involved in synthesizing different structural and signaling molecules that are critical for various cellular processes, e.g., lipid and glucose metabolism.^5^ However, the detection of FFAs with classical MALDI matrices and a lack of derivatization can generate ambiguous results since fatty acid signals can also represent fragments of intact lipid signals depending on the physiochemical nature of the matrix, along with the experimental conditions. Therefore, derivatization of FFAs with AMPP, which targets the −COOH end group, provides specificity for detection and imaging via MALDI-MSI.^47^ 2-Picolylamine and TMPA have previously been used to derivatize FFAs for detection within brain tissue sections using MALDI-MSI.^39,48^ AMPP/HATU derivatization enabled the detection of several saturated and unsaturated FFAs within rat brain tissue sections with MALDI-MSI (Figure 5).

**Figure 6:**
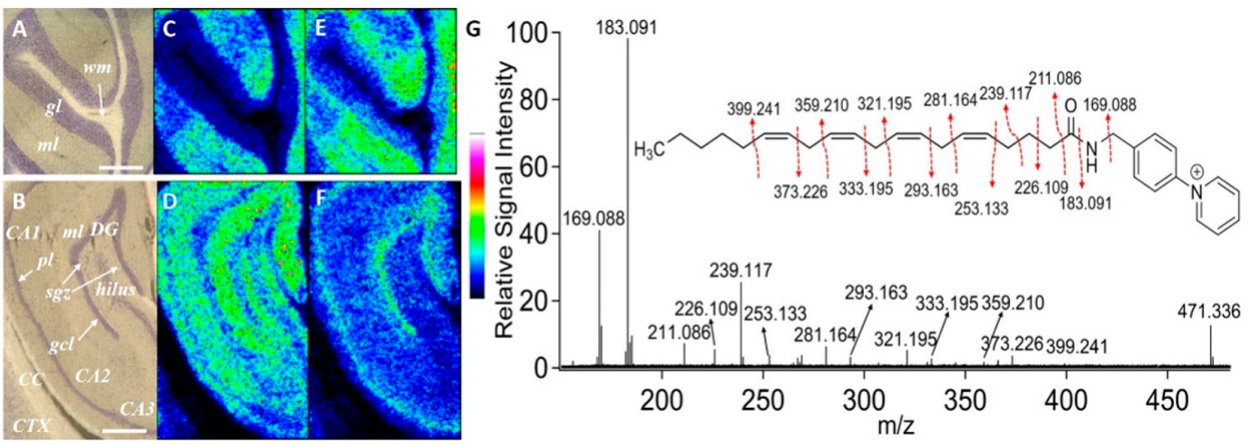
High-lateral resolution MALDI-MSI images of derivatized free fatty acids acquired from a rat brain tissue section. Optical images of the (A) cerebellum region and (B) hippocampal region from a coronal rat brain tissue section. Ion images reveal the distributions of AMPP-derivatized endogenous (C, D) arachidionic acid FA (20:4) (5,8,11,14) and (E, F) docosahexanoic acid FA (22:6). Lateral resolution, 20 μm. Data are shown using a rainbow scale (ion intensity scale) for optimal visualization. (G) MALDI-MS/MS spectrum obtained from rat brain tissue sections using MALDI-CID-FTICR from the precursor ion at *m*/*z* 471.336. Fragmentation pathways for AMPP-derivatized arachidionic acid have been proposed. CA, cornu ammonis; CC, corpus callosum; CTX, cortex; DG, dentate gyrus; pl, pyramidal layer; gcl, granule cell layer; ml, molecular layer; sgz, subgranular zone. Scale bars in panels (A) and (B) are 500 μm.

Further, we demonstrated that AMPP/HATU derivatization is relevant for high-lateral resolution (at 20 μm) MALDI-MSI of free arachidonic acid (AA) and docosahexanoic acid (DHA) within the rat cerebellum and hippocampus (Figure 6). MALDI ion images revealed that the distributions of AA and DHA are similar within the cerebellum (Figure 6C,E), whereas these molecules show distinct distributions within the hippocampus (Figure 6D,F). Both AA and DHA demonstrate abundant distribution on the molecular layer within the cerebellum (Figure 6C,E), while AA shows distinct distributions within the CA, molecular layer of the dentate gyrus, and subgranular zone within the dentate gyrus of the hippocampus (Figure 6D) and DHA shows distinct accumulation in the pyramidal and granular cell layers of the hippocampus (Figure 6F). Another advantage of AMPP derivatization is the possibility to determine double bond positions in FFAs via the production of structurally informative fragment ions during CID of AMPP-derivatized FFAs, as has already been demonstrated in previous LC–MS studies.^49^ Here, we demonstrated that it is possible to determine the double bond positions of AMPP-derivatized AA with on-tissue MS/MS using MALDI-CID-FTICR (Figure 6G).

### MALDI–MSI of a Primate Model of Parkinson’s Disease

**Figure 7:**
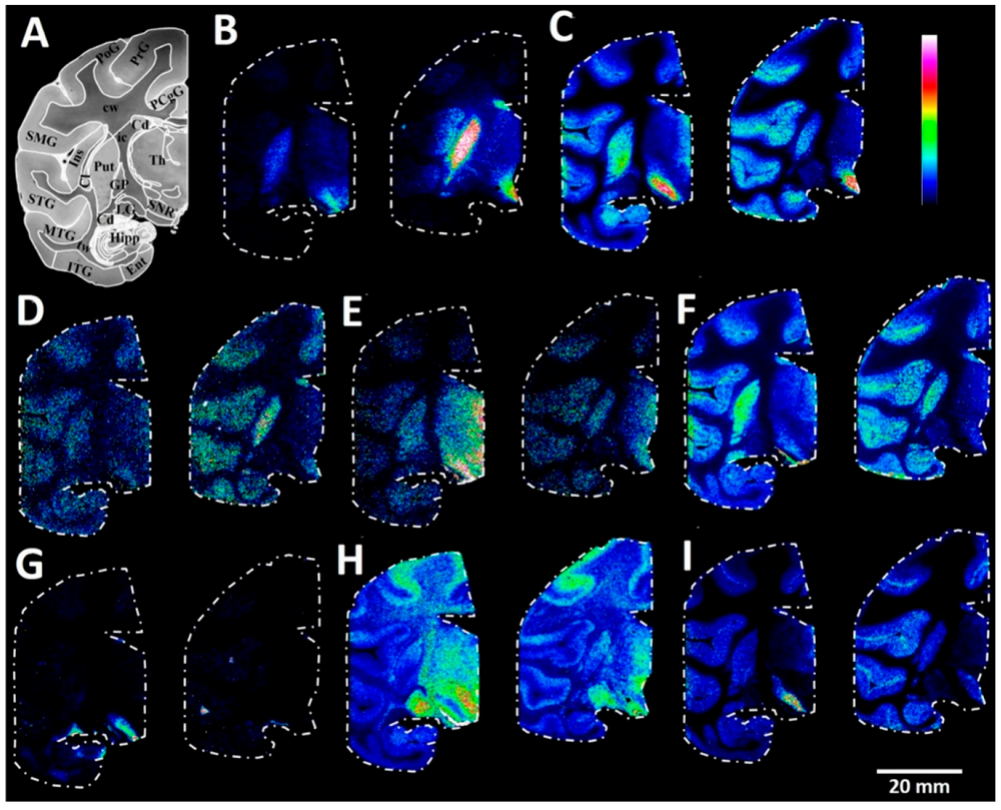
MALDI–MS images of carboxyl and aldehyde metabolites in the coronal region of primate control and Parkinson’s disease model brain tissue sections. (A) Brightfield image of a macaque brain tissue section at −7.5 mm ac with annotated brain regions. Ion images of (B) HVA (0–50%), (C) GABA (0–50%), (D) DOPAL (0–50%), (E) 5-HIAA (0–30%), (F) GHB (0–80%), (G) glucose/galactose (0–40%), (H) NAAG (0–10%), and (I) FA (20:5)-eicosapentaenoic acid (0–20%) from one MPTP-lesioned (left) and one control (right) animal at coronal level −7.5 mm ac. PoG, postcentral gyrus; PrG, precentral gyrus; PCgG, posterior singulate gyrus; SMG, supramarginal gyrus; STG, superior temporal gyrus; MTG, middle temporal gyrus; ITG, inferior temporal gyrus; Ent, entorhinal area; Hipp, hippocampus; Cd, caudate nucleus; Ins, insula; Cl, claustrum; Put, putamen; GP, globus pallidus, Th, thalamus; ic, internal capsule; LG, lateral geniculate nucleus; tw, temporal white matter; cw, cerebral white matter; SNR, substantia nigra pars reticulata. Lateral resolution, 150 μm.

PD is a neurodegenerative disease and the most common cause of movement disorders.^50^ While some information can be derived from neuropathological features, the etiology of PD, along with relevant pathogenetic and molecular mechanisms, remain enigmatic.^51^ MPTP-lesioned primate models include many of the clinical and pathological characteristics of PD.^52^ Therefore, we analyzed coronal brain tissue sections from one MPTP-lesioned and one control primate to illustrate the relevance of AMPP/HATU derivatization in studying PD (Figure 7A). We imaged certain catecholaminergic, serotonergic, and GABAergic metabolites with carboxyl and/or aldehyde functional groups in the molecular structure, e.g., HVA, GABA, DOPAL, 5-HIAA, and GHB (Figure 7B–F). Furthermore, we also imaged glucose/galactose, NAAG, and eicosapentaenoic acid, which revealed clear differences in specific brain regions of the MPTP-lesioned and control primate coronal brain tissue sections (Figure 7G–I).

As expected, we observed depletion of dopaminergic metabolites (HVA and DOPAL) in the caudate nucleus and putamen regions of the MPTP-lesioned primate brain (Figure 7B,D).^19^ MPTP-lesioned primate brain sections revealed elevated levels of the serotonergic neurotransmitter metabolite 5-HIAA in the hypothalamic regions and the substantia nigra reticulata Figure 7E.^19^ The MPTP-lesioned brain sections also demonstrated increased GABA levels in the globus pallidus and putamen (Figure 7C) and increased GHB levels in the putamen relative to the control brain sections^19^ (Figure 7F). AMPP/HATU derivatization also revealed clear region-specific changes in carboxyl- and aldehyde-containing metabolites, which had not yet been observed through mass spectrometry of primate brain tissue sections. For example, the MPTP-lesioned primate brain sections displayed higher levels of glucose/galactose and eicosapentaenoic acid in the substantia nigra reticulata than the control sections (Figure 7G,I). It was reported that the substantia nigra reticulata of MPTP-lesioned primate brains shows a significant decrease in glucose utilization,^53^ which is in line with the results of the present study. Elevated levels of eicosapentaenoic acid could be explained by a response against MPTP neurotoxin-induced oxidative stress within the substantia nigra. In line with this observation, the treatment of MPTP-lesioned animals with certain polyunsaturated fatty acids, including eicosapentaenoic acid, has demonstrated some degree of improvement against behavioral impairments, neurodegeneration, and inflammation within the brain.^54,55^ We also observed increased levels of NAAG within the thalamus region of MPTP-lesioned primate brain tissue sections relative to the control primate brain tissue sections (Figure 7H).

In conclusion, brain tissue sections from one control macaque and one MPTP-lesioned macaque provided a basis for comparing the distributions of numerous biologically important carboxyl and aldehyde metabolites across different brain regions with high mass accuracy. We were able to distinguish how MPTP-induced dopaminergic denervation alters the spatial distribution of certain molecules. This enabled us to demonstrate that dopamine degeneration causes effects in different brain regions, which is in accordance with the current understanding of PD pathophysiology.^19,28,53−55^

## Conclusions

We developed an on-tissue chemical derivatization and MALDI-MSI approach for the comprehensive mapping of carboxyls and aldehydes associated with important metabolic pathways within rodent and primate brain tissue sections. The AMPP derivatization reagent produced covalently charge-tagged molecules containing carboxylic acids (via a peptide coupling reaction) and aldehydes (via a Schiff base reaction) within one experiment. We evaluated eight different peptide coupling reagents, with HATU or HBTU emerging as the optimal reagents. The presented methodology was useful for detecting and imaging numerous carboxyl- and aldehyde-containing metabolites within various metabolic pathways, including the TCA cycle, fatty acid synthesis, glycolysis, and lipid peroxidation, as well as neurotransmitter and amino acid metabolism. Furthermore, AMPP/HATU derivatization is applicable for probing regional changes of metabolites within MPTP-administered and control primate brain tissue sections; the results revealed marked changes in the distributions of molecules within pathologically relevant brain regions, including hypothalamic regions, substantia nigra reticulata, putamen and globus pallidus, between the control and MPTP-administered brain tissue sections. This insight complements previous knowledge of the molecular mechanisms underlying MPTP-induced PD pathophysiology.

## Supporting Information Available

MALDI-CID-FTICR MS/MS and mass accuracy information on the identified metabolites. Comparison of the MALDI-FTICR-MSI ion images obtained from the consecutive tissue sections with 9-AA matrix and AMPP/HATU derivatization. Schematic of the MALDI-MSI ion images of the metabolites identified within TCA cycles and some of the associated metabolic pathways (PDF)
