# Supplementary material for: On-Tissue Chemical Derivatization for Comprehensive Mapping of Brain Carboxyl and Aldehyde Metabolites by MALDI–MS Imaging
Source: J Am Soc Mass Spectrom. 2023 Apr 13;34(5):836–46. doi: 10.1021/jasms.2c00336 (PMC10161219; doi:10.1021/jasms.2c00336)
Supplement: Supplementary file 1 — js2c00336_si_001.pdf [file js2c00336_si_001.pdf]

SUPPORTING INFORMATION

On-Tissue Chemical Derivatization for Comprehensive Mapping of Brain Carboxyl and Aldehyde Metabolites by MALDI–MS Imaging

Ibrahim Kaya[a], Luke S. Schembri[b], Anna Nilsson[a], Reza Shariatgorji[a], Sooraj Baijnath[a], Xiaoqun Zhang[c], Erwan Bezard[d], Per Svenningsson[c], Luke R. Odell[b*] and Per E. Andrén[a*]

a) Department of Pharmaceutical Biosciences, Spatial Mass Spectrometry, Science for Life Laboratory, Uppsala University, SE-75123 Uppsala, Sweden
b) Department of Medicinal Chemistry, Uppsala University, SE-75123 Uppsala, Sweden
c) Section of Neurology, Department of Clinical Neuroscience, Karolinska Institutet, SE-17177 Stockholm, Sweden.
d) Université de Bordeaux, Institut des Maladies Neurodégénératives, F-33000 Bordeaux, France

Corresponding Authors

Per E. Andrén

Department of Pharmaceutical Biosciences, Spatial Mass Spectrometry, Science for Life Laboratory, Uppsala University, BMC 591, SE-75124 Uppsala, Sweden

per.andren@farmbio.uu.se; +46-70 167 9334

Luke R. Odell

Department of Medicinal Chemistry, Uppsala University, BMC 591, SE-75124 Uppsala, Sweden

luke.odell@ilk.uu.se; +46-18 471 4297

Content

Supporting Information, Figure S1-S9 **page 2-10**

Supporting Information, Table S1 **page 11-12**

Supporting Information, References **page 12**

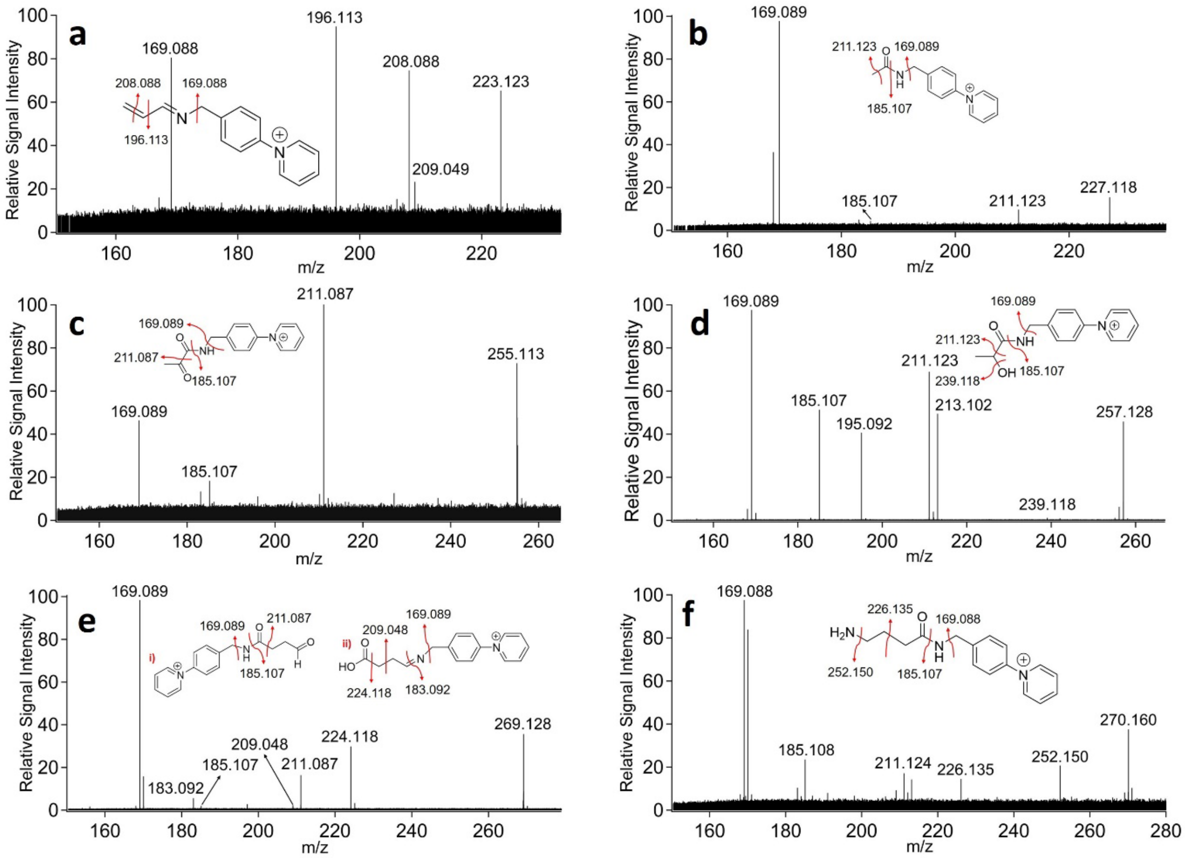

Supporting Information Figure S1. MS/MS spectra obtained from rat brain tissue sections using MALDI-CID-FTICR from the precursor ions at (a) *m/z* 223.123, (b) *m/z* 227.118, (c) *m/z* 255.113, (d) *m/z* 257.128, (e) *m/z* 269.128, and (f) *m/z* 270.160. Proposed fragmentation pathways for AMPP-derivatized (a) acrolein, (b) acetic acid, (c) pyruvic acid, (d) lactic acid, (e) succinic semialdehyde, and (f) γ-aminobutyric acid (GABA). Due to the isolation width of 1 *m/z* unit, additional isobaric and isomeric compounds could also be fragmented, giving rise to additional product ions.

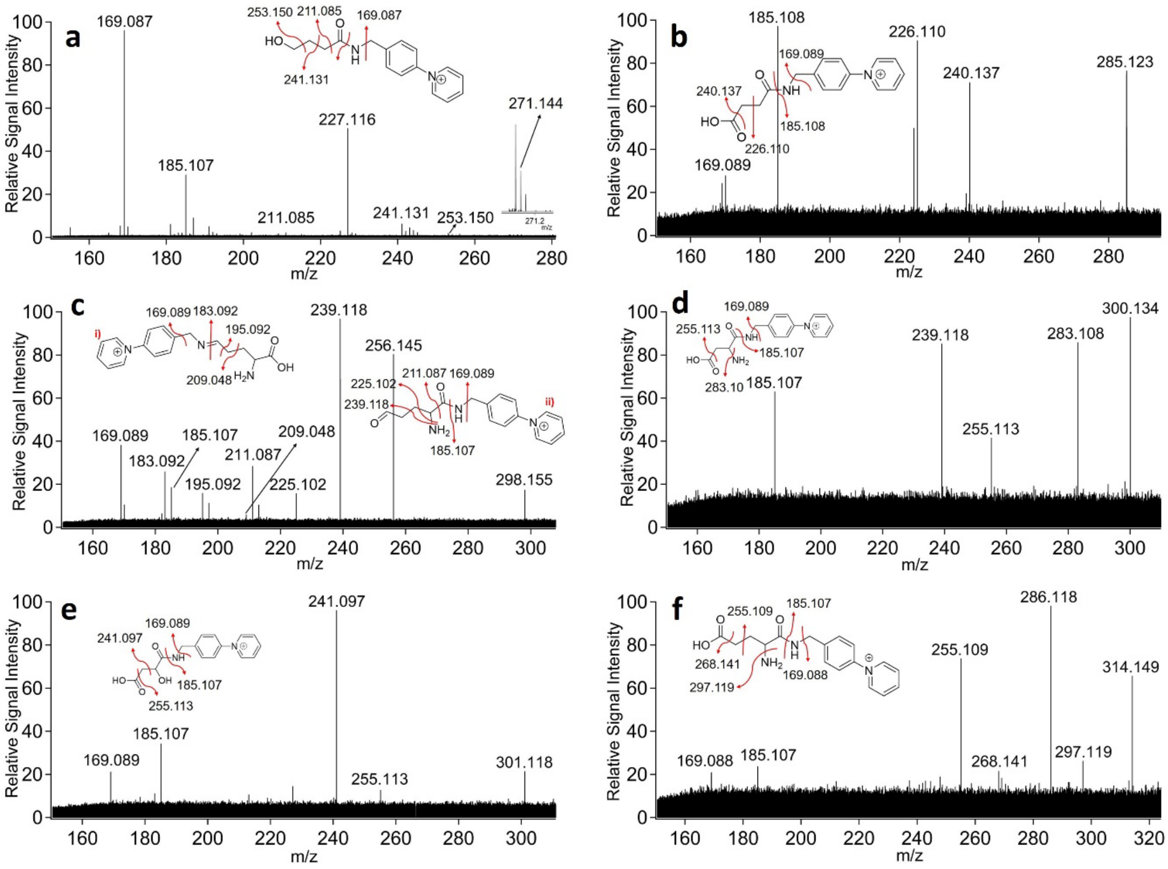

Supporting Information Figure S2. MS/MS spectra obtained from rat brain tissue sections using MALDI-CID-FTICR from the precursor ions at (a) *m/z* 271.144, (b) *m/z* 285.123, (c) *m/z* 298.155, (d) *m/z* 300.134, (e) *m/z* 301.118, and (f) *m/z* 314.149. Proposed fragmentation pathways for AMPP-derivatized (a) hydroxybutyric acid-GHB, (b) succinic acid, (c) glutamate semialdehyde, (d) aspartic acid, (e) malic acid, and (f) glutamate. Due to the isolation width of 1 *m/z* unit, additional isobaric and isomeric compounds could also be fragmented, giving rise to additional product ions.

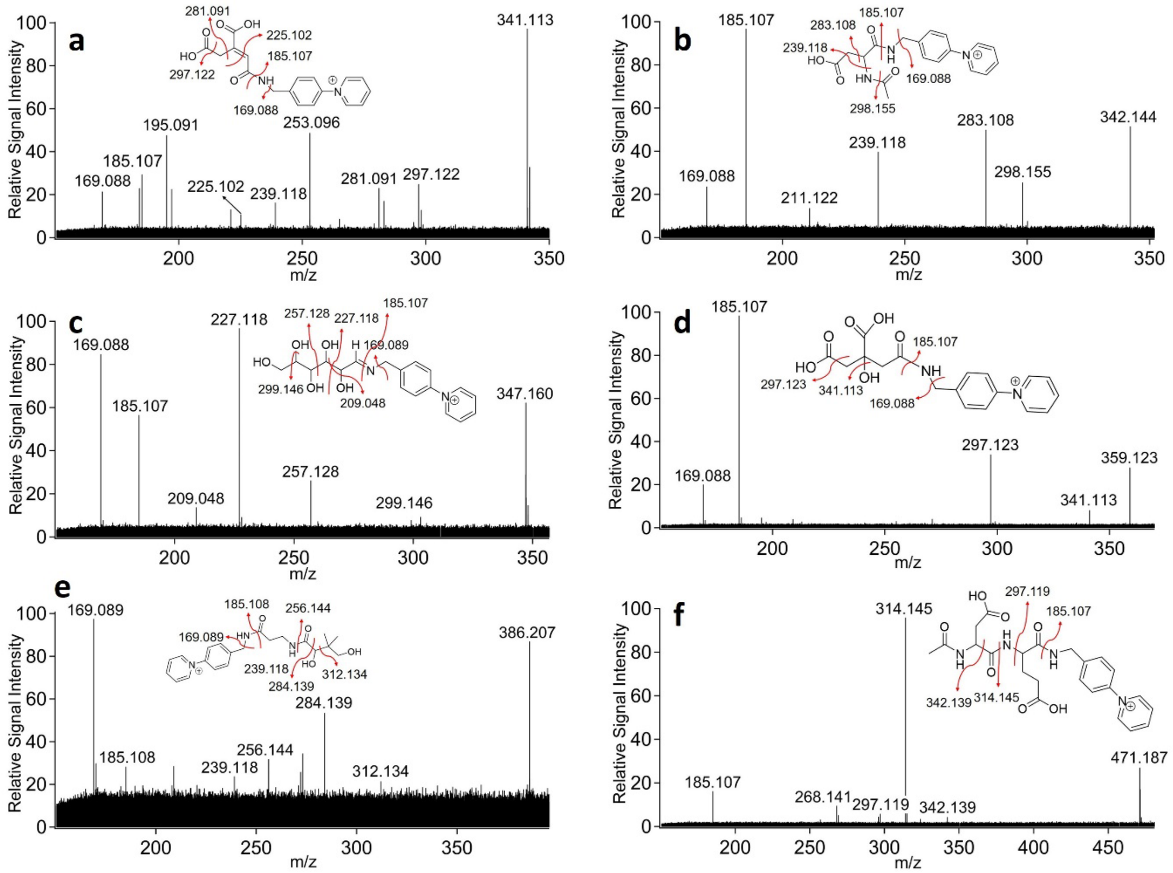

Supporting Information Figure S3. MS/MS spectra obtained from rat brain tissue sections using MALDI-CID-FTICR from the precursor ions at (a) *m/z* 341.113, (b) *m/z* 342.144, (c) *m/z* 347.160, (d) m/z 359.123, (e) *m/z* 386.207, and (f) *m/z* 471.187. Proposed fragmentation pathways for AMPP-derivatized (a) aconitic acid, (b) N-acetyl aspartic acid, (c) glucose/galactose, (d) citric acid/isocitric acid, (e) pantothenic acid, and (f) N-acetyl aspartyl glutamate (NAAG). Due to the isolation width of 1 *m/z* unit, additional isobaric and isomeric compounds could also be fragmented, giving rise to additional product ions.

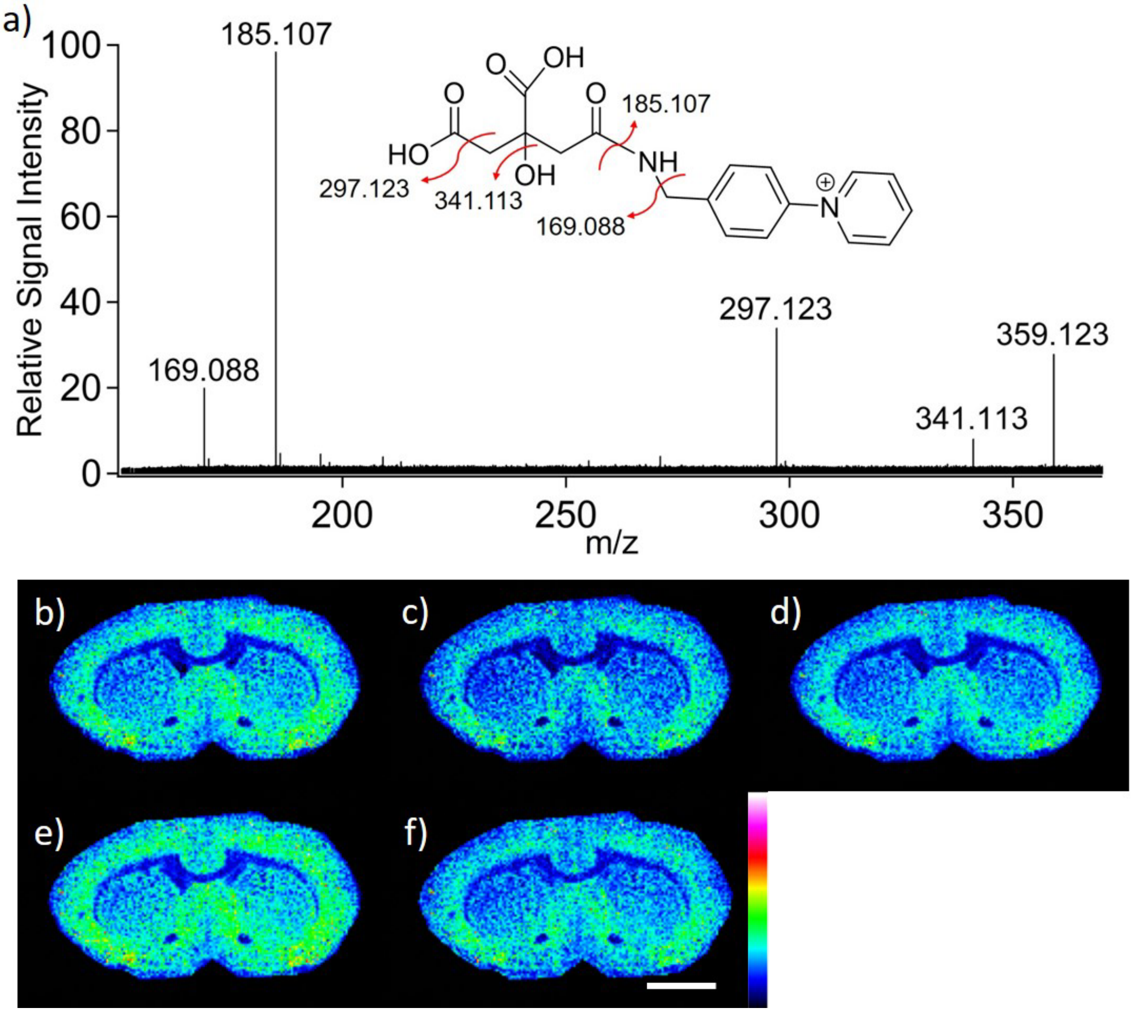

Supporting Information Figure S4. (a) MS/MS spectrum showing product ions supporting the assignment of citric acid/isocitric acid obtained from rat brain tissue sections using MALDI-CID-FTICR from the precursor ion at *m/z* 359.123. MALDI-MS/MS imaging of coronal rat brain tissue sections using MALDI-CID-FTICR reveals the distributions of the precursor and product ions (without normalisation) at (b) *m/z* 359.123, (c) *m/z* 341.113, (d) *m/z* 297.123, (e) *m/z* 185.107, and (f) *m/z* 169.088. Due to the isolation width of 1 *m/z* unit, additional isobaric and isomeric compounds could also be fragmented, giving rise to additional product ions. The scale bar is 3 mm.

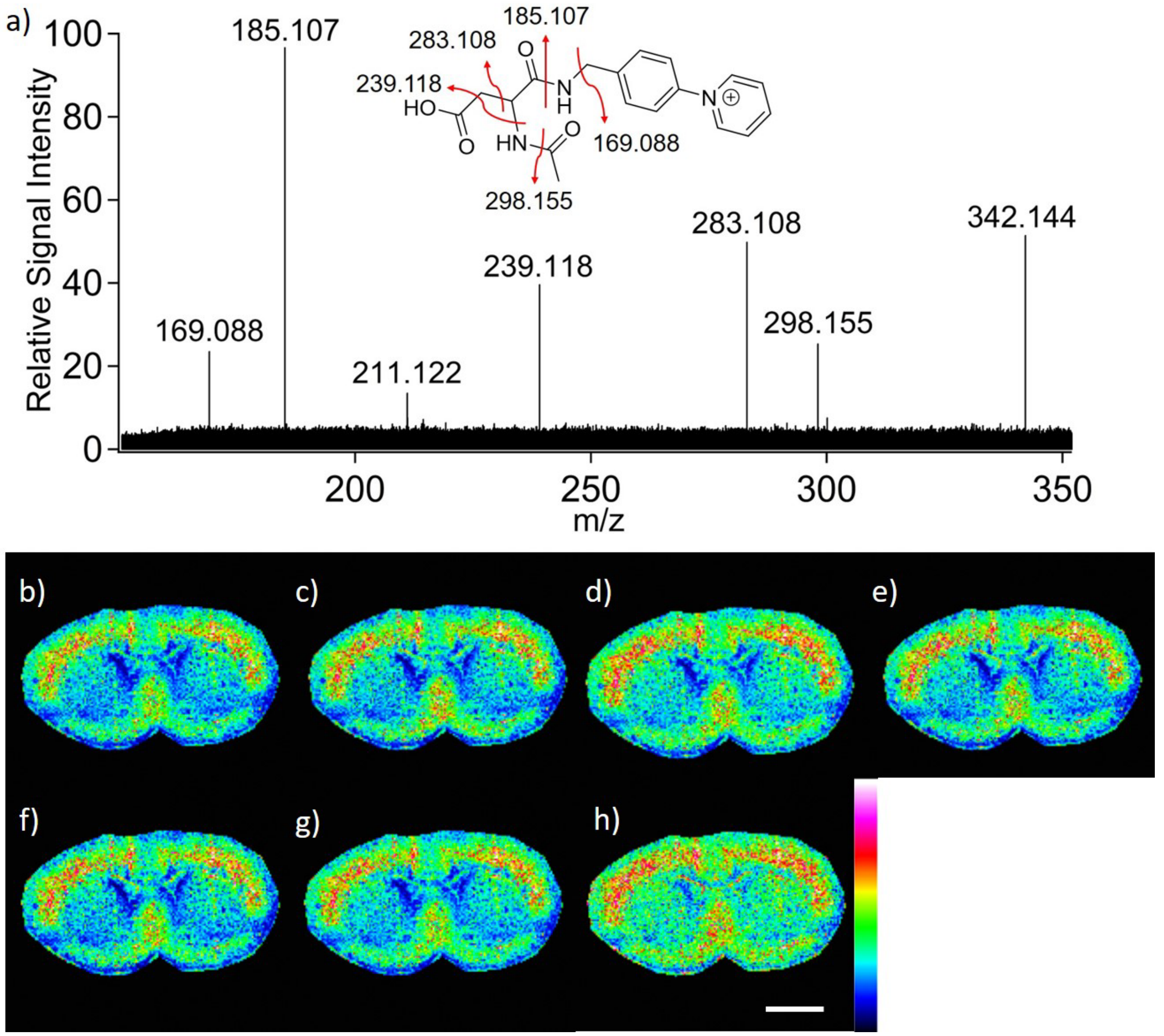

Supporting Information Figure S5. (a) MS/MS spectrum showing product ions supporting the assignment of *N*-acetyl aspartic acid obtained from rat brain tissue sections using MALDI-CID-FTICR from the precursor ion at *m/z* 342.144. MALDI-MS/MS imaging of coronal rat brain tissue sections using MALDI-CID-FTICR reveals the distributions of the precursor and product ions (without normalisation) at (b) *m/z* 342.144, (c) *m/z* 298.155, (d) *m/z* 283.108, (e) *m/z* 239.118, (f) *m/z* 211.122, (g) *m/z* 185.107, and (h) *m/z* 169.088. Due to the isolation width of 1 *m/z* unit, additional isobaric and isomeric compounds could also be fragmented, giving rise to additional product ions. The scale bar is 3 mm.

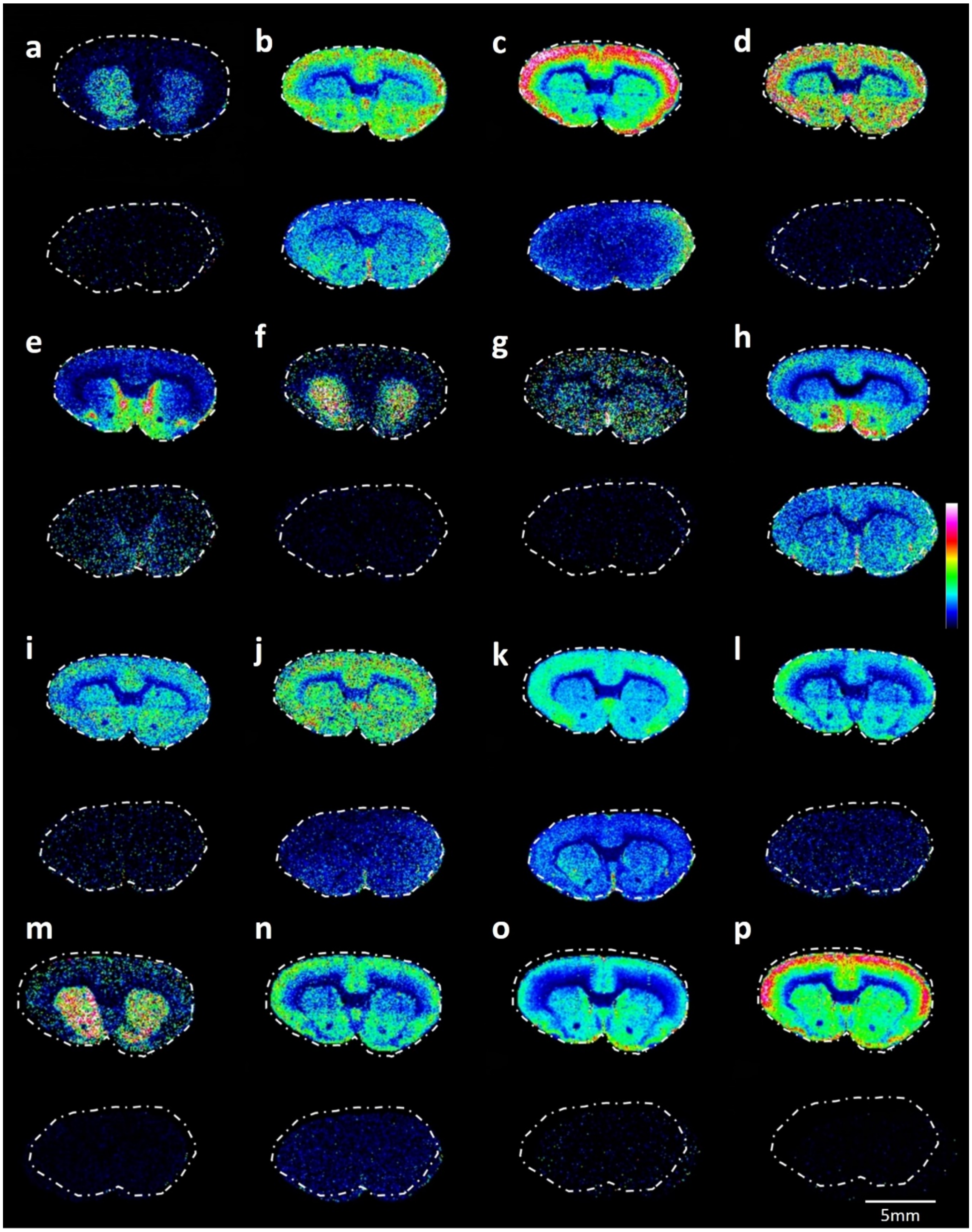

Supporting Information Figure S6. Comparative MALDI-MS images of metabolites within 6-OHDA-lesioned rat brain tissue with AMPP/HATU derivatization and a norharmane matrix (upper panel) versus a 9-AA matrix (lower panel). MALDI-MSI ion images of AMPP/HATU derivatized compounds in positive ion mode: (a) DOPAC, (b) aconitic acid, (c) succinic acid, (d) oxaloacetic acid, (e) pantothenic acid, (f) homovanilic acid (HVA), (g) 5-hydroxyindoleacetic acid (HIAA), (h) gamma-aminobutyric acid (GABA), (i) hydroxybutyric acid-GHB, (j) α-ketoglutaric acid, (k) citric acid/isocitric acid, (l) acetic acid, (m) DOPAL, (n) acrolein, (o) pentadecanal, and (p) heptadecanal. Data are shown using a rainbow scale (ion intensity scale) for optimal visualization. Lateral resolution, 100 μm.

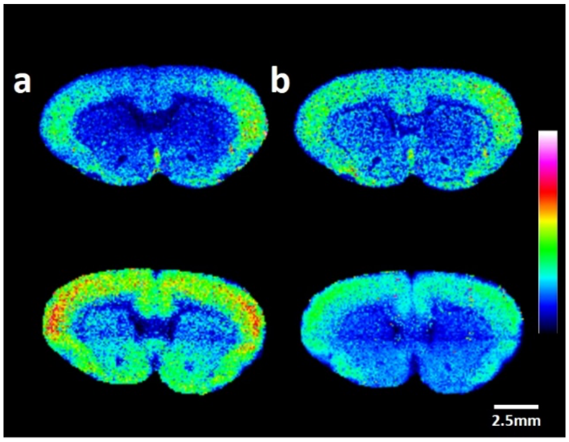

Supporting Information Figure S7. Comparative MALDI-MS images of (a) aspartic acid and (b) *N*-acetyl aspartic acid in 6-OHDA-lesioned rat brain tissue sections with a 9-AA matrix (upper panel) versus AMPP/HATU derivatization and a norharmane matrix (lower panel). Data are shown using a rainbow scale (ion intensity scale) for optimal visualization. Lateral resolution, 100 µm.

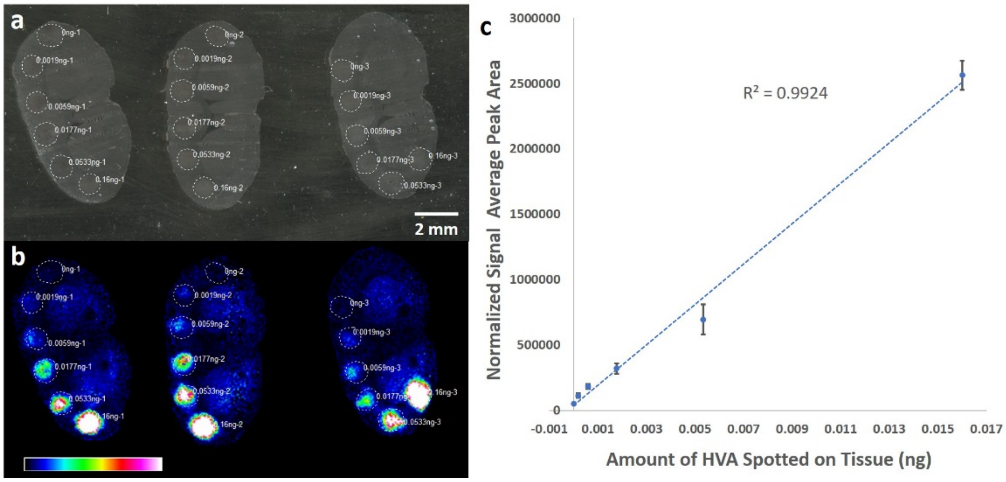

Supporting Information Figure S8. Linear calibration curve of HVA within the striatum of coronal rat brain tissue sections. (a) Brightfield images of three consecutive coronal rat brain tissue sections spotted with 0.10 µl of a HVA solution with the following concentrations: 0.16, 0.0533, 0.0177, 0.0059, 0.0019, and 0 ng/µl; the solution was spotted on the cortex of three consecutive coronal rat brain tissue sections. (b) MALDI-MS ion images of derivatized endogenous HVA (0-15%) and the spotted HVA solution (representing concentrations of 0-15%). (c) Linear calibration curve (spotted ng of HVA vs normalized signal average peak area) within the detectable range for HVA obtained by normalizing the HVA signals to HVA-d_5 signals in rat coronal brain tissue sections. Lateral resolution, 100 µm.

Evaluation of HVA Quantification with AMPP/HATU Derivatization and MALDI-MSI. We generated a linear calibration curve for HVA in rat coronal brain tissue sections using AMPP/HATU derivatization as follows. We prepared solutions (in 50% EtOH) at various concentrations of HVA, namely, 0.16, 0.0533, 0.0177, 0.0059, 0.0019, and 0 ng/µl, and then spotted 0.10 µl of each solution across the cortex of three consecutive coronal rat brain tissue sections (Figure S8a). Next, we sprayed 0.31 µg/ml of HVA-d_5 on tissue sections under the following conditions: nozzle temperature was set at 90°C, the reagent was sprayed pneumatically (6 psi of N2) in six passes at a linear velocity of 110 cm/min with 2 mm track spacing and a flow rate of 80µl/min; AMPP/HATU derivatization and MALDI-MSI were performed as indicated in the methods part. We detected endogenous HVA, as well as the spotted HVA down to a concentration of 0.00019 ng/µl, from the rat brain tissue sections (Figure S8b). We normalized the HVA signals to HVA-d_5 signals in SCiLS Lab (v. 2019a Pro, Bruker Daltonics) software and generated a linear calibration curve (Figure S7c) using the average peak area obtained from three consecutive replicates of each solution on three consecutive coronal rat brain tissue sections. Due to the presence of endogenous HVA within the cortex of rat brain tissue sections, we do not claim absolute quantification of the amount of HVA within rat brain tissue sections. However, the good linear response to our concentration range indicates that our method can be used to quantify certain metabolites within brain tissue sections.

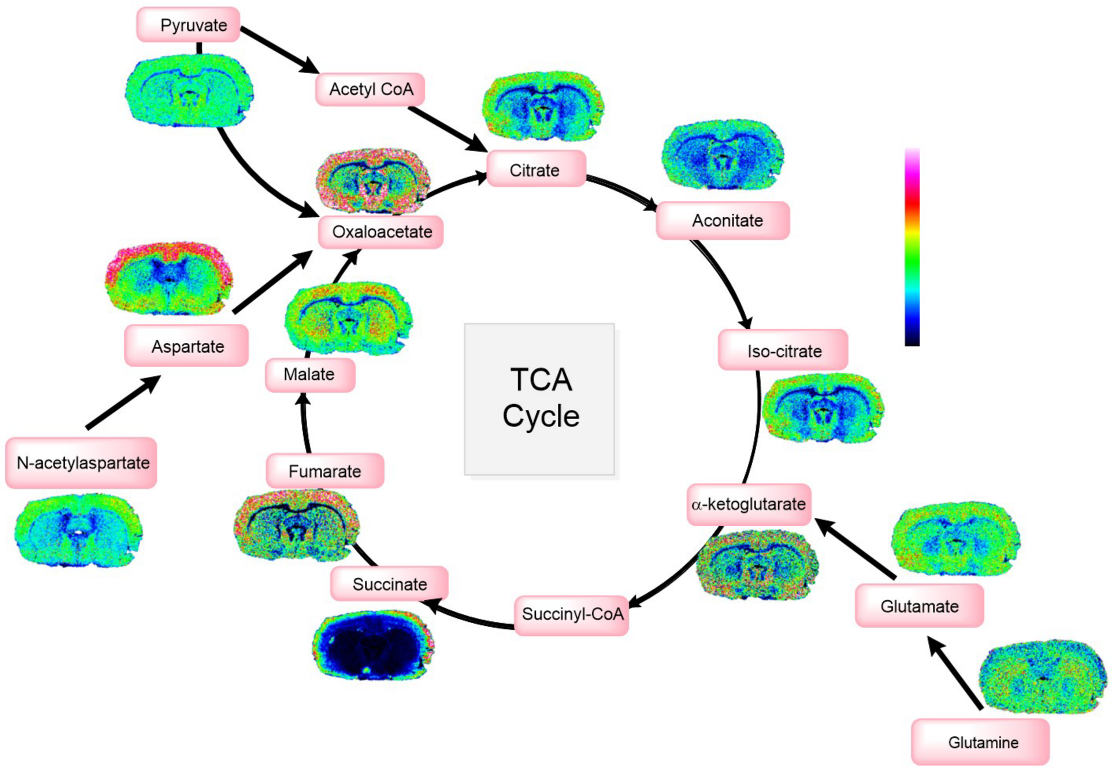

Supporting Information Figure S9. Representative ion images of identified metabolites within the TCA cycle and some associated metabolic pathways from rat brain tissue sections obtained using AMPP/HATU derivatization and MALDI-FTICR-MSI. Data are shown using a rainbow scale (ion intensity scale) for optimal visualization. Lateral resolution, 100 μm.

Supporting Information Table S1. AMPP-derivatized aldehyde and carboxyl metabolite species were observed within brain tissue sections, including several neurotransmitters, amino acids and the associated metabolites, dipeptides, TCA cycle metabolites, glycolysis metabolites, reactive aldehydes, free fatty acids and fatty aldehydes. Free fatty acid (FFA) species, including essential fatty acids, were identified based on the fragment ions indicating that they had been derivatized with AMPP; the species were then annotated as the most likely fatty acid species based on abundance within brain tissues according to HMDB information (www.hmdb.ca) and previously published reports.[1] However, we were able to identify arachidonic acid (AA) and docosahexaenoic acid (DHA) based on specific double bond positional information obtained via on-tissue MS/MS. For fatty aldehydes, we obtained initial MS/MS data to validate that the detected compounds had been derivatized with AMPP and fatty aldehydes were identified based on the primary hits from HMDB (www.hmdb.ca). Neurotransmitter species, including DOPAL, DOPAC, HIAA, HIAL, HVA, and GABA, were determined based on mass accuracy, specific distributions within brain tissue sections, and alterations in 6-OHDA-lesioned coronal rat brain tissue sections and MPTP macaque brain tissue sections, as previously validated by our group[2] (see also SI Figure S6). We obtained MS/MS data for several metabolites directly from rat and/or macaque brain tissue sections (SI Figure S1-3). However, additional isobaric and isomeric compounds could also be fragmented within the same MS/MS analysis. ʹIn the case that there were multiple primary hits for a molecule identified based on accurate mass, the compound with the highest likelihood of existing within brain tissue was selected.

Metabolite	Formula	M+AMPP Formula	M	M-H	M+H	M+AMPP	M+AMPP observed	ppm error
Citric Acid/Isocitric Acid	C6H8O7	C18H19N2O6	192.027003	191.019727	193.034279	359.123763	359.1238	-0.10
GABA	C4H9NO2	C16H20N3O	103.063329	102.056053	104.070605	270.160089	270.1604	-1.15
FA(20:4)-Arachidionic Acid	C20H32O2	C32H43N2O	304.24023	303.232954	305.247506	471.33699	471.3368	0.40
Hydroxyglutaric Acid*	C5H8O5	C17H19N2O4	148.037173	147.029897	149.044449	315.133933	315.1342	-0.85
Fumaric Acid*	C4H4O4	C16H15N2O3	116.010959	115.003683	117.018235	283.107719	283.108	-0.99
Succinic Acid	C4H6O4	C16H17N2O3	118.026609	117.019333	119.033885	285.123369	285.12371	-1.20
Malic Acid	C4H6O5	C16H17N2O4	134.021523	133.014247	135.028799	301.118283	301.11852	-0.79
Oxaloacetic Acid*	C4H4O5	C16H15N2O4	132.005873	130.998597	133.013149	299.102633	299.1029	-0.89
α-ketoglutaric Acid*	C5H6O5	C17H17N2O4	146.021523	145.014247	147.028799	313.118283	313.1188	-1.65
Lactic Acid	C3H6O3	C15H17N2O2	90.031694	89.024418	91.03897	257.128454	257.1288	-1.35
FA(22:6)-Docosahexanoic acid	C22H32O2	C34H43N2O	328.24023	327.232954	329.247506	495.33699	495.33651	0.97
DOPAL	C8H8O3	C20H19N2O2	152.047344	151.040068	153.05462	319.144104	319.14427	-0.52
Aspartic Acid	C4H7NO4	C16H18N3O3	133.037508	132.030232	134.044784	300.134268	300.13445	-0.61
FA(18:1)-Oleic Acid	C18H34O2	C30H45N2O	282.25588	281.248604	283.263156	449.35264	449.35254	0.22
FA(18:2)-Linoleic Acid*	C18H32O2	C30H43N2O	280.24023	279.232954	281.247506	447.33699	447.33676	0.51
FA(18:3)-Linolenic Acid	C18H30O2	C30H41N2O	278.22458	277.217304	279.231856	445.32134	445.3212	0.31
FA(16:0)-Palmitic Acid	C16H32O2	C28H43N2O	256.24023	255.232954	257.247506	423.33699	423.33687	0.28
FA(16:1)-Palmitoleic Acid	C16H30O2	C28H41N2O	254.22458	253.217304	255.231856	421.32134	421.3212	0.33
FA(18:0)-Steraic Acid	C18H36O2	C30H47N2O	284.27153	283.264254	285.278806	451.36829	451.3678	1.09
FA(22:4)-Docosatetranoic Acid	C22H36O2	C34H47N2O	332.27153	331.264254	333.278806	499.36829	499.36785	0.88
FA(22:5)-Docosapentaenoic acid	C22H34O2	C34H45N2O	330.25588	329.248604	331.263156	497.35264	497.35222	0.84
FA(20:1)-Eicosenoic Acid*	C20H38O2	C32H49N2O	310.28718	309.279904	311.294456	477.38394	477.38353	0.86
FA(20:5)-Eicosapentaenoic Acid*	C20H30O2	C32H41N2O	302.22458	301.217304	303.231856	469.32134	469.321	0.72
Glutamate	C5H9NO4	C17H20N3O3	147.053158	146.045882	148.060434	314.149918	314.15013	-0.67
Glutamine*	C5H10N2O3	C17H21N4O2	146.069142	145.061866	147.076418	313.165902	313.1661	-0.63
HVA	C9H10O4	C21H21N2O3	182.057909	181.050633	183.065185	349.154669	349.15484	-0.49
DOPAC	C8H8O4	C20H19N2O3	168.042259	167.034983	169.049535	335.139019	335.1393	-0.84
Pyruvic Acid	C3H4O3	C15H15N2O2	88.016044	87.008768	89.02332	255.112804	255.1131	-1.16

5-HIAA	C10H9NO3	C22H20N3O2	191.058243	190.050967	192.065519	358.155003	358.15513	-0.35
N-Acetylaspartic Acid	C6H9NO5	C18H20N3O4	175.048072	174.040796	176.055348	342.144832	342.1451	-0.78
Aconitic Acid	C6H6O6	C18H17N2O5	174.016438	173.009162	175.023714	341.113198	341.1134	-0.59
Acetic Acid	C2H4O2	C14H15N2O	60.021129	59.013853	61.028405	227.117889	227.11832	-1.90
Glucose/Galactose	C6H12O6	C18H23N2O5	180.063388	179.056112	181.070664	347.160148	347.16037	-0.64
Pantothenic Acid	C9H17NO5	C21H28N3O4	219.110673	218.103397	220.117949	386.207433	386.20743	0.01
5-HIAL	C10H9NO2	C22H20N3O	175.063329	174.056053	176.070605	342.160089	342.16051	-1.23
HNE*	C9H16O2	C21H27N2O	156.11503	155.107754	157.122306	323.21179	323.212	-0.65
Malondialdehyde/Pyruvaldehyde*	C3H4O2	C15H15N2O	72.021129	71.013853	73.028405	239.117889	239.1183	-1.72
Acrolein	C3H4O	C15H15N2	56.026215	55.018939	57.033491	223.122975	223.1234	-1.90
Creatine*	C4H9N3O2	C16H20N5O	131.069477	130.062201	132.076753	298.166237	298.16657	-1.12
Glycine*	C2H5NO2	C14H16N3O	75.032028	74.024752	76.039304	242.128788	242.1292	-1.70
Gln-Gln*	C10H18N4O5	C22H29N6O4	274.12772	273.120444	275.134996	441.22448	441.22414	0.77
Gly-Phe/Phe-Gly*	C11H14N2O3	C23H25N4O2	222.100442	221.093166	223.107718	389.197202	389.1972	0.01
NAAG	C11H16N2O8	C23H27N4O7	304.090665	303.083389	305.097941	471.187425	471.18706	0.77
beta-ctryl glutamate*	C11H15NO10	C23H26N3O9	321.069596	320.06232	322.076872	488.166356	488.16601	0.71
Pentadecanal	C15H30O	C27H41N2	226.229666	225.22239	227.236942	393.326426	393.3264	0.07
Pentadecenal	C15H28O	C27H39N2	224.214016	223.20674	225.221292	391.310776	391.31068	0.25
Heptadecanal	C17H34O	C29H45N2	254.260966	253.25369	255.268242	421.357726	421.3576	0.30
Hexadecanal	C16H32O	C28H43N2	240.245316	239.23804	241.252592	407.342076	407.3419	0.43
Octadecanal	C18H36O	C30H47N2	268.276616	267.26934	269.283892	435.373376	435.3732	0.40
Tetradecanal	C14H28O	C26H39N2	212.214016	211.20674	213.221292	379.310776	379.3107	0.20
Alanine/Sarcosine*	C3H7NO2	C15H18N3O	89.047678	88.040402	90.054954	256.144438	256.1449	-1.80
Lysine*	C6H14N2O2	C18H25N4O	146.105528	145.098252	147.112804	313.202288	313.2024	-0.36
Leucine/Isoleucine*	C6H13NO2	C18H24N3O	131.094629	130.087353	132.101905	298.191389	298.19169	-1.01
Glutamate-semialdehyde	C5H9NO3	C17H20N3O2	131.058243	130.050967	132.065519	298.155003	298.1553	-1.00
Hydroxybutyric Acid-GHB	C4H8O3	C16H19N2O2	104.047344	103.040068	105.05462	271.144104	271.1445	-1.46
Succinic Semialdehyde	C4H6O3	C16H17N2O2	102.031694	101.024418	103.03897	269.128454	269.1288	-1.29

REFERENCES

1. Wu, Q.; Comi, T. J.; Li, B.; Rubakhin, S. S.; Sweedler, J. V., On-tissue derivatization via electrospray deposition for matrix-assisted laser desorption/ionization mass spectrometry imaging of endogenous fatty acids in rat brain tissues. *Anal. Chem.* **2016,** *88* (11), 5988-5995.
2. Shariatgorji, M.; Nilsson, A.; Fridjonsdottir, E.; Vallianatou, T.; Källback, P.; Katan, L.; Sävmarker, J.; Mantas, I.; Zhang, X.; Bezard, E., Comprehensive mapping of neurotransmitter networks by MALDI–MS imaging. *Nat. Methods* **2019,** *16* (10), 1021-1028.
